# Homochiral *D*_4_-symmetric metal–organic cages from stereogenic Ru(II) metalloligands for effective enantioseparation of atropisomeric molecules

**DOI:** 10.1038/ncomms10487

**Published:** 2016-02-03

**Authors:** Kai Wu, Kang Li, Ya-Jun Hou, Mei Pan, Lu-Yin Zhang, Ling Chen, Cheng-Yong Su

**Affiliations:** 1 MOE Laboratory of Bioinorganic and Synthetic Chemistry, State Key Laboratory of Optoelectronic Materials and Technologies, Lehn Institute of Functional Materials, School of Chemistry and Chemical Engineering, Sun Yat-Sen University, Guangzhou 510275, China; 2 State Key Laboratory of Applied Organic Chemistry, Lanzhou University, Lanzhou 730000, China

## Abstract

Absolute chiral environments are rare in regular polyhedral and prismatic architectures, but are achievable from self-assembly of metal–organic cages/containers (MOCs), which endow us with a promising ability to imitate natural organization systems to accomplish stereochemical recognition, catalysis and separation. Here we report a general assembly approach to homochiral MOCs with robust chemical viability suitable for various practical applications. A stepwise process for assembly of enantiopure *ΔΔΔΔΔΔΔΔ*- and *ΛΛΛΛΛΛΛΛ-*Pd_6_(RuL_3_)_8_ MOCs is accomplished by pre-resolution of the *Δ*/*Λ*-Ru-metalloligand precursors. The obtained Pd–Ru bimetallic MOCs feature in large *D*_4_-symmetric chiral space imposed by the predetermined Ru(II)-octahedral stereoconfigurations, which are substitutionally inert, stable, water-soluble and are capable of encapsulating a dozen guests per cage. Chiral resolution tests reveal diverse host–guest stereoselectivity towards different chiral molecules, which demonstrate enantioseparation ability for atropisomeric compounds with *C*_2_ symmetry. NMR studies indicate a distinctive resolution process depending on guest exchange dynamics, which is differentiable between host–guest diastereomers.

## Abstract (web summary)

Homochiral molecular capsules offer potential applications in chiral separation and stereospecific catalysis. Here, by pre-resolution of *Δ/Λ*-metalloligand precursors, the authors are able to assemble enantiopure supramolecular cages capable of stereoselective host-guest behaviour.

## Graphical abstract

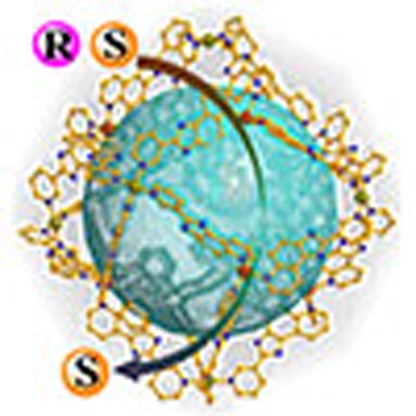

The design and synthesis of discrete nanoscale metal–organic cages/containers (MOCs) with specific configurations and cavities applying directional bridging ligands and geometrically prefixed metals is emerging as an appealing topic in recent supramolecular coordination chemistry^1^,^2^,^3^. Among this, the controlled assembly of enantiopure chiral cages is of special importance because of their potential applications in stereoselective recognition, catalysis and enzyme mimics^4^,^5^,^6^,^7^,^8^,^9^,^10^,^11^. Since the chiral space in regular polyhedra only rarely presents in snub dodecahedron and snub cube (all other Platonic, Archimedean, prismatic and antiprismatic solids are achiral)^12^,^13^, assembly of chiral polyhedral MOCs is usually achieved by introducing stereogenic centres into the faces, edges or vertices of a polyhedron to remove inversion and mirror symmetries. In this way, a number of homochiral MOCs of *T*-symmetry^14^,^15^,^16^,^17^,^18^,^19^ have been constructed, whereas the chiral MOCs of *O*-symmetry or higher were proved to be more formidable because of more possible stereoisomers and the demand to transmit single chirality from more subcomponents^20^,^21^. In principle, the chirality of an MOC can be generated either by the organic stereocentres (such as chiral tetrahedral C*) or the metal stereogenic centres. The latter strategy provides a versatile platform for stereochemistry of MOCs because the plentiful metal coordination geometries can afford innumerable stereogenic metal centres for assembly of chiral structures even from achiral components in a supramolecular sense^22^,^23^,^24^. The overall MOC symmetry can be restricted or reduced by the stereochemical coupling between metal centres. For example, transfer of stereoconfiguration information between vertices of a tetrahedron enables absolute assembly^24^ of homoconfigurational *ΔΔΔΔ*- or *ΛΛΛΛ*-cages based on the stereogenic tris-chelate metal centres^15^,^16^,^17^. However, the lability of metal–ligand exchange often causes enantiomerization between opposite enantiomers^25^, and racemic mixture cannot be prevented during the assembly process. Resolution of the enantiopure product usually has to be accomplished with the aid of chiral auxiliaries to form diastereomers, and stabilization of the dynamic metal centre often needs synergistic effect^15^,^16^,^17^.

An alternative way to construct stable and robust homochiral MOCs based on the stereogenic metal centres is to design a metalloligand^26^ containing a stereoconfigurationally inert metal centre in lieu of the C* stereocentre in organic ligand. Formation of MOCs by virtue of various metalloligands has been achieved in many excellent lines of works^27^,^28^,^29^,^30^,^31^,^32^, in which spontaneous resolution and geometric isomerism were observed^33^,^34^,^35^, yet construction of enantiopure MOCs from predetermined chiral metalloligands remains unexplored. On the basis of the well-known stereochemistry of *D*_3_-symmetric [Ru(bpy)_3_]^2+^- or [Ru(phen)_3_]^2+^-type compounds, which are widely explored in DNA interactions, asymmetric catalysis and supramolecular chiral assemblies^36^,^37^,^38^,^39^,^40^, we initiated the design of [Ru(phen)_3_]^2+^-type metalloligand for homochiral MOC self-assembly^26^. Since the stereoconfiguration of such tri-chelate Ru-octahedral centres is substitutionally inert and stable in solution assembly and crystallization process, we expect that the predetermined chirality of the Ru metalloligands can direct the assembly of homochiral MOCs with sufficient stability in practical applications. Although stereoselective recognition and catalysis using chiral hosts has been well established^14^,^15^,^16^,^17^,^18^,^19^,^41^,^42^, enantioseparation of racemic guest molecules by means of homochiral coordination cages remains a challenge. Only a few examples are known to achieve moderate to good diastereoselectivity^43^,^44^,^45^,^46^, thus urging an extensive study to solve the common problems in this field; for example, (a) efficient resolution of enantiopure cages, (b) effective stabilization of cage stereochemistry and (c) high guest inclusion capacity (more than three guests per host). Herein we report a general approach to assemble homochiral MOCs without post resolution based on the pre-resolved stereogenic Ru-octahedral centres, offering huge cages capable of large amounts of guest encapsulation (>10 guests per host). Specifically, stereoselective separation of atropisomeric molecules rather than C*-based chiral compounds is achieved, and a dynamic resolution process based on differentiable guest exchange by formation of diastereomers is proposed.

## Results

### Assembly of enantiopure MOCs

**Figure 1:**
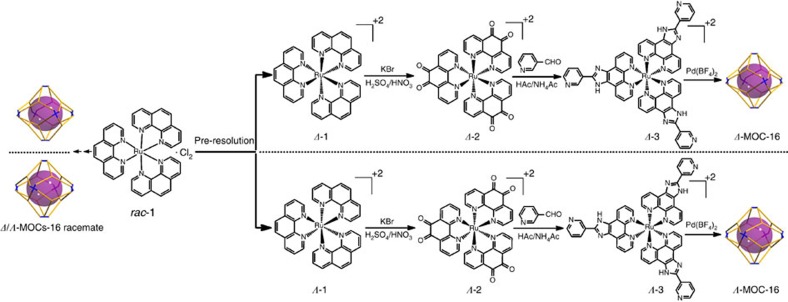
Assembly procedures. Formation of racemic *Δ*/*Λ*-Pd_6_(RuL_3_)_8_ cages (*rac*-*Δ*/*Λ*-MOCs-16) from mixed precursors, and stepwise syntheses of enantiopure *Δ*- and *Λ*-Pd_6_(RuL_3_)_8_ cages (*Δ-/Λ*-MOCs-16) from pre-resolved *Δ-*3 and *Λ*-3 metalloligands.

We have previously assembled heteronuclear *Δ*/*Λ*-Pd_6_(RuL_3_)_8_ MOCs racemate (hereafter assigned as ***rac*****-*****Δ*****/*****Λ*****-MOCs-16**, Fig. 1) from the racemic RuL_3_ metalloligands (***rac*****-*****Δ*****/*****Λ*****-3**), which show the shape of an octahedron (defined by Pd_6_ centres) or a rhombic dodecahedron (defined by Pd_6_Ru_8_ centres)^26^. It was noted that the cage assembly proceeded in a homochiral manner, with each individual **MOC-16** integrating the same handed ***Δ*****-** or ***Λ*****-3** enantiomers to display either *ΔΔΔΔΔΔΔΔ* or *ΛΛΛΛΛΛΛΛ* homoconfigurations, indicative of strong cooperative stereochemical coupling between the metal centres^14^,^15^,^16^,^17^,^18^,^19^,^22^,^23^,^24^ to direct the absolute self-organization^24^ and exclusive formation of single homochiral ***Δ**-* or***Λ*****-MOC-16**. However, thus assembled chiral ***Δ**-* and***Λ*****-MOCs** co-crystallize simultaneously to give racemic products that are not ready for practical applications.

To make use of these homochiral cages, we started chiral resolution from well-established pre-resolution of *Δ*/*Λ*-[Ru(phen)_3_]^2+^ precursors and developed a pair of enantiomeric triangular metalloligands incorporating fixed chiral octahedral Ru(II) centres and pyridyl (Py) terminals ready for assembly of enantiopure *Δ*- and *Λ*-Pd_6_(RuL_3_)_8_ MOCs separately. As shown in Fig. 1 and described in detail in Supplementary Figs 1–10, racemic *Δ*/*Λ*-[Ru(phen)_3_]^2+^ was first resolved into a pair of enantiomers (***Δ***- and ***Λ*****-1**) in good yields using K_2_[Sb_2_{(+)-tartrate}_2_]·3H_2_O as chiral induction agent, and then oxidized into *Δ-* and *Λ*-[Ru(Phendione)_3_]^2+^ (***Δ***- and ***Λ*****-2**). With the aid of chiral shift reagent Eu((+)tfc)_3_, the enantiopurity was tested to be 94.8% for ***Δ*****-1** and 95.3% for ***Λ*****-1** (ref. 47). The absolute configurations of the two pairs of ***Δ*****-/*****Λ*****-1** and ***Δ**-***/*****Λ*****-2** enantiomers have been well established by the single-crystal structural analyses (Supplementary Figs 1 and 2), which are in excellent agreement with the experimental resolution and syntheses. The phase purity of the bulk products of ***Δ*****-/*****Λ*****-1** and ***Δ**-***/*****Λ*****-2** enantiomers has also been verified using the powder X-ray diffraction measurements (Supplementary Figs 1 and 2). Further reaction of ***Δ*****-** and ***Λ*****-2** with 3-pyridinecarboxaldehyde afforded a pair of stereogenic bulky *Δ-* and *Λ*-RuL_3_ metalloligands (***Δ*****-** and ***Λ*****-3**), and, finally, the coordination assembly of ***Δ*****-** and ***Λ*****-3** enantiomers with Pd^2+^ ions unambiguously resulted in a pair of homochiral *Δ*- and *Λ*-Pd_6_(RuL_3_)_8_ cages, namely ***Δ*****-MOC-16** and ***Λ*****-MOC-16**, respectively. ^1^H NMR spectra of two optically pure ***Δ*****-/*****Λ*****-MOCs-16** enantiomers give well-resolved proton patterns basically identical to previously reported racemic ***rac*****-*****Δ*****/*****Λ*****-MOCs-16** (Supplementary Fig. 8), showing distinguishable H resonance between the protons inside and outside cage (Supplementary Fig. 9). The ^1^H-^1^H-COSY and high-resolution mass spectrometry (HR-ESI-TOF-MS) have also been performed to verify formation of Pd_6_(RuL_3_)_8_ cage structures (Supplementary Fig. 10).

**Figure 2:**
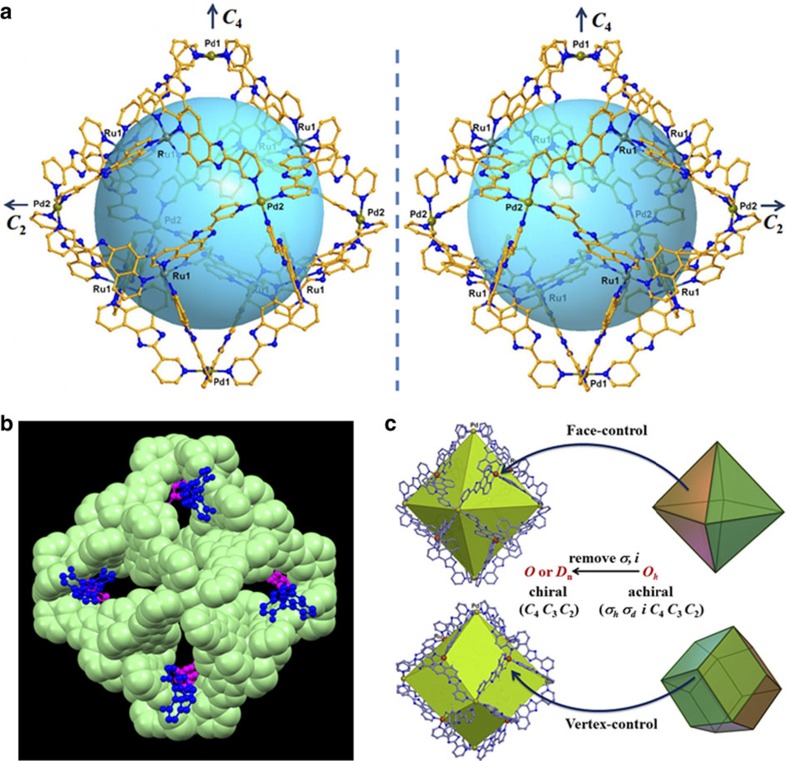
Crystal structures. (**a**) A pair of *D*_4_-symmetric homochiral *Δ-* and *Λ*-MOCs-16 showing *ΔΔΔΔΔΔΔΔ* and *ΛΛΛΛΛΛΛΛ* configurations of eight RuL_3_ metalloligands whereas *ΛΛΛΛΛΛ*- and *ΔΔΔΔΔΔ*-configurations of six Pd-Py_4_ subcomponents. (**b**) A *Δ*-MOC-16 cage (in space-filling mode) capturing eight *S*-BINOL guests (in ball-and-stick mode) on the windows pockets. (**c**) The demonstration how to form *D*_4_ *Λ*-MOC-16 from *O*_h_ regular polyhedra by introducing eight *Λ*-3 metalloligands on the faces of an octahedron, or, on the *C*_3_ vertices of a rhombic dodecahedron, to reduce molecular symmetry, and further direct *Δ*-arrangement of four pyridyl rings around six vertices of Pd centres.

The absolute configurational arrangement of the ***Δ*****-** or ***Λ*****-3** metalloligands in ***Δ*****-MOC-16** or ***Λ*****-MOC-16**, respectively, has been undoubtedly established by the single-crystal analyses (Supplementary Fig. 3). The single crystals of ***Δ*****-MOC-16** and ***Λ*****-MOC-16** were grown from their MeCN solutions in the presence of *S*-BINOL and *R*-BINOL, respectively, as absolute structural reference compounds for further authentication of the crystal chirality. Both ***Δ*****-MOC-16** and***Λ-*****MOC-16** crystallize in the chiral space groups *I*422. In ***Δ*****-MOC-16**, eight ***Δ*****-3** metalloligands are assembled by six square-coordinative Pd^2+^ ions to form Pd_6_(RuL_3_)_8_ cage with the *ΔΔΔΔΔΔΔΔ* homoconfigurations (Fig. 2a). The crystal is packed by the identical ***Δ*****-MOC-16** cages in together with *S*-BINOL molecules, giving rise to enantiopure product with the absolute chirality exactly according to the chiral ***Δ*****-3** metalloligands and reference *S*-BINOL used in syntheses and crystal growth. In contrast, ***Λ*****-MOC-16** integrates eight ***Λ*****-3** metalloligands and six Pd^2+^ ions to form Pd_6_(RuL_3_)_8_ cage with the *ΛΛΛΛΛΛΛΛ* homoconfigurations (Fig. 2a), and co-crystallizes with *R*-BINOLs to result in enantiopure crystals. For both ***Δ*****-MOC-16** and ***Λ-*****MOC-16**, the powder X-ray diffraction patterns of the bulk samples well match those of the single-crystal simulations, indicating satisfactory phase purity (Supplementary Fig. 3).

Careful examination of the crystal structures of ***Δ*****-/*****Λ-*****MOCs-16** enantiomers reveals that the cage molecule possesses crystallographically imposed *D*_4_ symmetry (Fig. 2 and Supplementary Fig. 3). If regarding the cage as a pseudo-octahedron, the *C*_4_ axis passes two vertices occupied by Pd1 ions, while two pairs of Pd2 ions are located on the *C*_2_ axes. Therefore, the cage symmetry may be considered to degrade from chiral *O*-symmetry owing to disposition of the same handed Ru-stereocentres on eight faces of octahedron, or, on eight *C*_3_ vertices of rhombic dodecahedron to impose *ΔΔΔΔΔΔΔΔ* or *ΛΛΛΛΛΛΛΛ* homoconfigurations in ***Δ*****-MOC-16** and ***Λ*****-MOC-16**, respectively. In another word, the assembly of the homochiral ***Δ**-***/*****Λ*****-MOCs-16** enantiomers proceeds in a way of octahedral face-control or rhombic dodecahedral vertex-control, thus removing inversion *i* and mirror *σ* symmetries to turn an achiral *O*_*h*_ group into a chiral *D*_4_ group (Fig. 2c). Furthermore, the stereoconfigurations around six Pd^2+^ vertices are also induced by the fixed Ru-stereocentres. In ***Δ*****-MOC-16**, six Pd-Py_4_ subcomponents are all in *Λ*-configurations, with the four Py rings showing anticlock fan-like arrangement and *vice versa* in ***Λ*****-MOC-16**. In contrast to other completely labile coordination cages^14^,^15^,^16^,^17^,^18^,^19^,^20^,^21^,^22^,^23^,^24^, in the present cases, the stereoconfiguration around the Pd^2+^ corner is fixed by inserting Ru-stereocentres and cage integrity; therefore, enantiomerization through labile Pd-ligand exchange is inhibited for the whole cage. It is worth mentioning that eight *S*-BINOLs are captured by a ***Δ*****-MOC-16**, or reversely, eight *R*-BINOLs by a ***Λ*****-MOC-16**, on its window pockets but not completely into its cavity (Fig. 2b and Supplementary Figs 3a,b) probably because the crystallization takes place in the MeCN solution where hydrophobic effect is absent and the host–guest inclusion behaviour is different from that in aqueous medium discussed below (*vide infra*).

**Figure 3:**
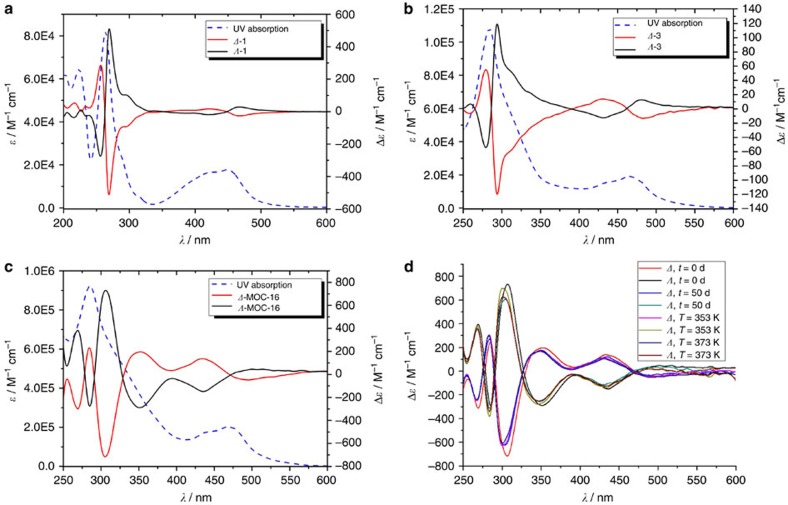
CD and UV spectra. CD (solid lines) and ultraviolet (dotted lines). (**a**) *Δ*- and *Λ*-1 in MeCN, (**b**) *Δ*- and *Λ*-3 in DMSO, (**c**) *Δ*- and *Λ*-MOCs-16 in H_2_O and (**d**) the stereochemical stability depending on time and temperature.

The circular dichroism (CD) spectra were also employed to monitor the whole synthetic and assembly processes to confirm that the absolute chirality of the starting *Δ-*/*Λ*-[Ru(phen)_3_]^2+^ precursors were well preserved all the way down (Fig. 3 and Supplementary Fig. 11). In MeCN solution, *Δ-*/*Λ*-[Ru(phen)_3_]^2+^ mainly presents three absorption peaks at 225, 265 and 450 nm (Fig. 3a), with the first two corresponding to the n–π* and π–π* transitions of phen groups, while the last one originating from the metal-to-ligand charge transfer (MLCT) transition between Ru^2+^ and phen ligands. All three absorption bands are reflected in the corresponding CD spectra of the resolved ***Δ*****-1** and ***Λ*****-1** with the middle peak at 265 nm, giving the most prominent CD signal. Taking ***Δ*****-1** as an example, the same tendency of first negative and second positive Cotton effect from longer to shorter wavelength in the three CD bands is in accordance with the *Δ*-type octahedral chirality established for the Ru^2+^ coordination centre^48^ and *vice versa* for the ***Λ*****-1** compound. For the rest three pairs of enantiomers, because of the cutoff effect of the solvents (dimethylsulphoxide (DMSO) or H_2_O), the CD signals corresponding to the absorption at 225 nm were not fully presented; however, the other bands were clearly detected in the whole synthetic process, preserving the same chirality attributes for the same series of enantiomers (***Δ*****-1**, **2**, **3**, **MOC-16** versus *Λ*-**1**, **2**, **3**, **MOC-16**, respectively). Furthermore, because of the accumulation effect (eightfold in ***Δ**-* or ***Λ*****-MOC-16** compared with ***Δ*****-** or ***Λ*****-3**) of multiple chiral Ru centres in one entity in the final enantiopure ***Δ**-***/*****Λ*****-MOCs-16**, a remarkable increase in CD signal intensities was observed for ***Δ*****-** or ***Λ*****-MOC-16** (Δ*ɛ*=∼720 M^−1 ^cm^−1^) in comparison with ***Δ*****-** or ***Λ*****-3** (Δ*ɛ*=∼120 M^−1 ^cm^−1^). Optical rotation tests also manifested the absolute configurations in ***Δ*****-** and ***Λ*****-MOCs-16** (***Δ***, [α]^20^_D_=−266°; ***Λ***, [α]^20^D=272°, *c*=0.5, H_2_O). From these CD studies we see that the stereochemistry of octahedral Ru centres is robust enough to survive all reaction conditions, exactly in agreement with the observations of chirality preservation in crystallographic study. The stereochemical stability of ***Δ*****-** and ***Λ*****-MOCs-16** has also been testified against heating and longtime stay in solution (Fig. 3d), confirming that the absolute chirality of each enantiomeric ***Δ**-* and ***Λ*****-MOC-16** is well retained on heating to 373 K and staying in solution for 50 days. Such a stable and substitutionally inert nature of stereogenic Ru centres plays a key role in fixing absolute chirality of ***Δ*****-** and ***Λ*****-MOCs-16**, despite intrinsic dynamics of Pd^2+^ centres subject to metal–ligand exchange, thereof paving the way for utilization of these enantiopure cages in, for example, stereoselective catalysis and separation.

### Stereoselective separation of racemic guests

**Figure 4:**
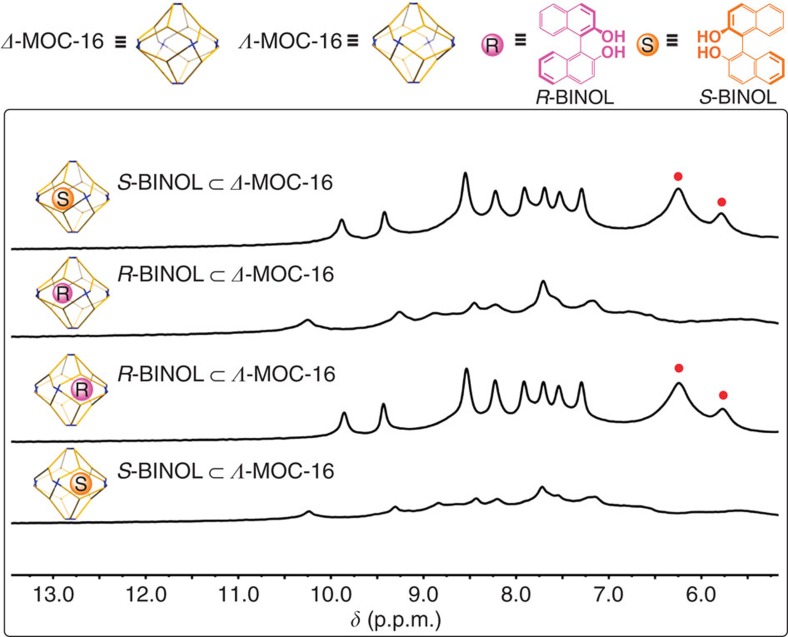
^1^H NMR enantiodifferentiation experiments. Sequestration of enantiomeric *R*- or *S*-BINOL guests by homochiral *Δ*- or *Λ*-MOCs-16 (*d*_6_-DMSO/D_2_O=1/5, 298 K). Red circles denote signals of encapsulated guests.

In an attempt to test enantioseparation ability of ***Δ*****-/*****Λ*****-MOC-16** cages, we selected two types of racemic organic molecules, one carrying a chiral C* centre and the other characteristic of *C*_2_-symmetric chirality (Table 1). The host–guest inclusion examined by ^1^H NMR in the D_2_O system revealed that all chiral molecules can be well encapsulated by the **MOC-16** host owing to hydrophobic effect (Supplementary Figs 12–16), showing typical upfield shift of guest protons and further splitting of cage protons^26^. Moreover, the host–guest stereochemical relationship between enantiomeric ***Δ*****-/*****Λ*****-MOCs-16** and *R*-/*S*-BINOLs has been examined using ^1^H NMR enantiodifferentiation experiments, where two pairs of host–guest diastereomers, namely *S*-BINOL**⊂*****Δ*****-MOC-16**, *R*-BINOL**⊂*****Δ*****-MOC-16** and *S*-BINOL**⊂*****Λ*****-MOC-16**, *R*-BINOL**⊂*****Λ*****-MOC-16**, and two pairs of host–guest enantiomers, namely *S*-BINOL**⊂*****Δ*****-MOC-16**, *R*-BINOL**⊂*****Λ*****-MOC-16** and *R*-BINOL**⊂*****Δ*****-MOC-16**, *S*-BINOL⊂***Λ*****-MOC-16**, are formed. As shown in Fig. 4, the solution dynamics is obviously distinguishable between the diastereomeric pairs, while that between the enantiomeric pairs is similar^41^. This means the homochiral ***Δ*****-** and ***Δ*****-MOC-16** cages are able to recognize and differentiate *R*- and *S*-BINOL enantiomeric guests in solution because of their diastereomeric host–guest relationship. As a consequence, the chiral resolution of racemic molecules was carried out by ***Δ*****-** and ***Λ*****-MOCs-16** separately in pure D_2_O solution based on either a homogeneous or a heterogeneous method (see details in Methods or Supplementary Methods). The resolved guests were determined using high-performance liquid chromatography (HPLC) with enantiomeric excess (*ee*) averaged from three parallel experiments (Table 1 and Supplementary Figs 17–24).

The resolution results unveil that the homochiral ***Δ**-* or ***Λ*****-MOCs-16** have rather poor stereoselectivity towards chiral compounds containing C* stereocentres. As seen in Table 1, no obvious resolution effect can be detected for naproxen, 1-(1-naphthyl)ethanol and benzoin, despite ^1^H NMR-proved inclusion of these guests by the host **MOC-16** (Supplementary Figs 14–16). However, through the same separation process, a pair of *R*-/*S*-BINOL atropisomers was successfully resolved, with the *ee* values reaching 34% or more by ***Δ*****-/*****Λ*****-MOCs-16**. Relatively low enantioseparation results were obtained for *R*-/*S*-3-Br-BINOL racemate; however, the resolution effect was greatly improved for the chiral discrimination of *R*-/*S*-6-Br-BINOL enantiomers. By applying ***Δ*****-MOC-16**, the resolved product contains 77% of *R*-isomer and 23% of *S*-isomer, giving an *ee* value of 54%. Surprisingly, an *ee* value up to 62% was obtained by ***Λ*****-MOC-16** with the product dominant in *S*-isomer. Similar enantioseparation ability of ***Δ*****-/*****Λ*****-MOCs-16** was able to extend to another kind of atropisomeric compound *R*-/*S*-spirodiol, exhibiting the same host–guest stereoselectivity. The ***Δ*****-MOC-16** got 34% predominance of *R*-isomer, while a higher *ee* value of 44% was obtained for *S*-isomer by ***Λ*****-MOC-16**. In general, ***Δ*****-MOC-16** shows a preferable stereoselectivity towards *R*-isomer, while ***Λ*****-MOC-16** prefers *S*-isomer for all chiral guests of *C*_2_ symmetry. The higher-resolution effect from *S*-isomer**⊂*****Λ*****-MOC-16** inclusion than from *R*-isomer**⊂*****Δ*****-MOC-16** inclusion is unexpected, probably owing to the slight difference in optical attribute based on their optical rotation tests. To the best of our knowledge, such a preferable enantiorecognition of chiral guests with *C*_2_ symmetry has not been observed before for cage compounds, and the enantioseparation ability of ***Δ*****-/*****Λ*****-MOCs-16** reaches high level within the known chiral organic and coordination cages^43^,^44^,^45^,^46^,^49^. In addition, the adequate solubility of **MOCs-16** in water (2.6 g per 100 ml) makes it convenient to implement enantioseparation either in a homogeneous two-phase way (*Method I:* organic-water transfer as shown in Supplementary Fig. 25) or simply in a heterogeneous suspension way (*Method II:* solid-solution transfer as described in Methods). In comparison with the normally insoluble metal–organic frameworks for chiral separation, the water solubility of **MOCs-16** offers advantages by using the hydrophobic effect to transfer water-insoluble organic guests into the aqueous phase, and the guest transformation between the organic-water phases is easy to accomplish. Extraction of the resolved chiral guests from the water phase of ***Δ*****-/*****Λ*****-MOCs-16** readily leads to recovery of the empty cages, which can be reused for the next runs of chiral resolution without a significant loss of the enantioseparation ability as tested by four cycles of *R/S*-6-Br-BINOL resolution with ***Δ*****-MOCs-16** (*ee* 51–57%, Supplementary Table 1). On the other hand, chiral resolution test of *R*/*S*-6-Br-BINOL racemate within ***Δ*****-MOC-16** and ***Λ*****-MOC-16** using 10 times the amount of host and guest indicates that the enantioselectivity is retained almost the same for the scaling up separation (*ee* 55 and 60%).

### Resolution process study

**Figure 5:**
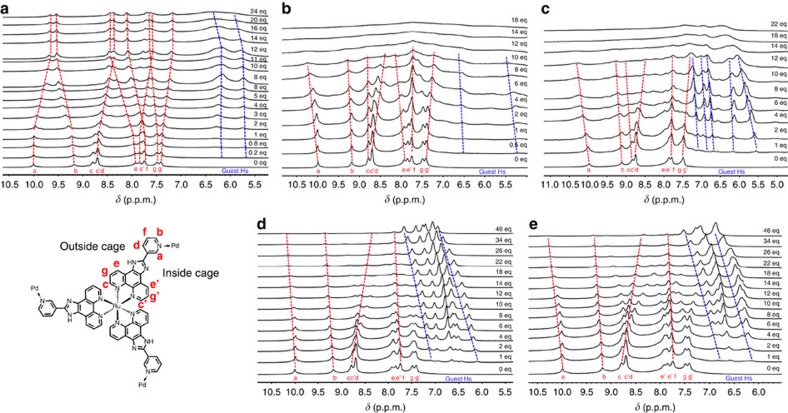
^1^H NMR titration in DMSO-*d*_6_/D_2_O=1/5. (**a**) *R*-BINOL⊂*Λ*-MOC-16 at 298 K, (**b**) *S*-BINOL⊂*Λ*-MOC-16 at 298 K, (**c**) *S*-BINOL⊂*Λ*-MOC-16 at 353 K, (**d**) *S*-1-(1-naphthyl)ethanol⊂*Λ*-MOC-16 at 298 K and (**e**) *S*-1-(1-naphthyl)ethanol⊂*Δ*-MOC-16 at 298 K.

To further understand the host–guest interactions for insight into the resolution mechanism, ^1^H NMR titration was performed in an attempt to acquire association constants^42^ for the pairs of host–guest diastereomers. However, the experimental results obviously reveal intricate host–guest solution dynamics (Fig. 5 and Supplementary Figs 26–28). Since the **MOC-16** cage has a huge molecular size (3.3 × 3.3 nm) and cavity (2895 Å^3^ based on VOIDOO calculations) where a large amount of guests could be hosted (for example, 18 Phen guests per host)^26^, the proton signals of both cage and guests are generally broadened and poorly resolved because of slow rotational diffusion and dynamics typical of large molecules, thereof preventing us from quantitative study with regard to thermodynamic or kinetic details by using the known methods for relatively simple host–guest systems (usually more than three guests per host)^50^,^51^,^52^,^53^. Nevertheless, it is evident that titration of enantiopure ***Λ*****-MOC-16** (or ***Δ*****-MOC-16**) with *R*- and *S*-BINOL guests of *C*_2_ symmetry undergoes remarkably different host–guest interaction processes, showing distinguishable guest inclusion behaviours for *R*- and *S*-BINOL atropisomers as demonstrated in Fig. 5a,b. This is in agreement with the observation from above-mentioned ^1^H NMR enantiodifferentiation experiments because of the formation of a pair of host–guest diastereomers *R*-BINOL⊂***Λ*****-MOC-16** and *S*-BINOL⊂***Λ*****-MOC-16**. In contrast, titration of ***Δ*****-** and ***Λ*****-MOC-16** cages with the same C*-chiral *S*-1-(1-naphthyl)ethanol guest, which should also give a pair of diastereomers *S*-1-(1-naphthyl)ethanol⊂***Δ*****-MOC-16** and *S*-1-(1-naphthyl)ethanol⊂***Λ*****-MOC-16**, just results in rather similar ^1^H NMR chemical shift patterns (Fig. 5d,e), indicating that the homochiral ***Λ*****-MOC-16** (or ***Δ*****-MOC-16**) cage exhibits the same guest inclusion behaviours for *R*- and *S*-enantiomeric guests carrying C* stereocentres.

As demonstrated in Fig. 5, stepwise inclusion of *R*-BINOLs by ***Λ*****-MOC-16** at 298 K causes inverse chemical shifts of cage protons, with those inside the cage moving upfield while those outside the cage moving downfield (Fig. 5a). The guest protons appear as severely broadened doublet and remain almost unmoved up to 12 guest inclusion. Addition of more than 12 equivalent *R*-BINOLs shows little influence on cage protons, but leads to downfield shift and further broadening of guest protons. These results suggest that at least 12 *R*-BINOLs are encapsulated inside the cage, and further guest uptake may speed up dynamic exchange. Inclusion of guests inside the cage is also supported with ^1^H-^1^H-COSY and NOESY measurements (Supplementary Fig. 26). For comparison, inclusion of *S*-BINOLs at 298 K does not lead to distinct bidirectional shifts of cage protons, while the resonance of guests is even broadened and becomes poorly visible together with the host protons on inclusion of more than 10 *S*-BINOLs (Fig. 5b). To observe guest signals more clearly, titration at a higher temperature 353 K was performed (Fig. 5c), which presents better resolved guest resonances but basically same overall chemical shift patterns as observed at 298. Therefore, similar host–guest interacting manners may be expected at these temperatures (*vide infra*). It is notable that the guest signals display a continuously downfield shift, characteristic of fast guest exchange. These NMR observations imply more dynamic host–guest interactions for *S*-BINOL⊂***Λ*****-MOC-16** inclusion compared with *R*-BINOL⊂***Λ*****-MOC-16** inclusion at the room temperature. Broadening of H resonance is indicative of slow and restricted molecular rotation and tumbling^54^,^55^ as well as of a comparable guest exchange rate with the NMR timescale. For the *S*-BINOL⊂***Λ*****-MOC-16** system, faster guest exchange dynamics may present, showing averaged influence on host protons either inside or outside. When the cage cavity is getting fulfilled (∼12 guest per cage), the overall host–guest dynamics is slowed down to make NMR unable to discriminate resonating frequency. In contrast, guest exchange in the *R*-BINOL⊂***Λ*****-MOC-16** system is slow enough at room temperature, thus showing distinguishable impact on host protons inside and outside. Such a guest dynamic difference between two host–guest diastereomers may account for the intrinsic factor that determines the enantioseparation ability of homochiral ***Δ*****-** or ***Λ*****-MOCs-16** towards racemic *R*/*S*-BINOLs. On the contrary, titration of ***Δ*****-** and ***Λ*****-MOCs-16** with *S*-1-(1-naphthyl)ethanol guests shows a similar guest exchange dynamics (Fig. 5d,e), where fast guest exchange is evident for both host–guest diastereomers. This may explain why the homochiral ***Δ*****-/*****Λ*****-MOCs-16s** are unable to discriminate *R*/*S*-stereomers carrying opposite C* stereocentres. Although a confinement effect of a cage is usually expected to enhance the intrinsic chirality of the C* guests, discriminable stereoselectivity was not observed for the present C* molecules because of fast guest exchange.

**Figure 6:**
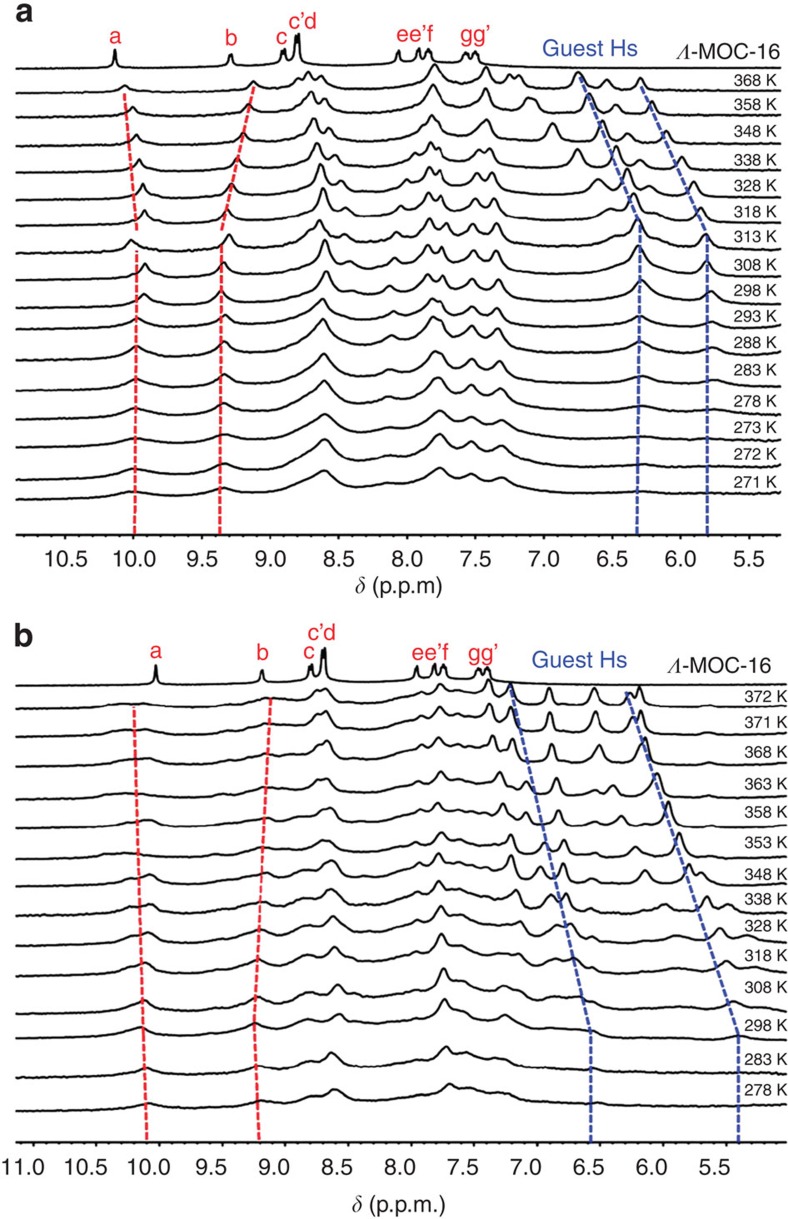
VT ^1^H NMR study of guest inclusion dynamics in solvent DMSO-d_6_/D_2_O=1/5. (**a**) ***R*****-BINOL⊂***Λ*-MOC-16. (**b**) ***S*****-BINOL⊂***Λ*-MOC-16. Red lines show host protons, while blue lines show guest protons.

Variable-temperature ^1^H NMR study has been carried out to testify the above-proposed resolution process (Fig. 6 and Supplementary Fig. 29). It is clear that, for both *R*-BINOL⊂***Λ*****-MOC-16** and *S*-BINOL⊂***Λ*****-MOC-16** diastereomers, heating boosts guest dynamics and accelerates guest exchange, with two broadened signals getting better resolved and moving constantly downfield to approach free guests. This kind of host–guest solution dynamics might be comparable to the NMR-observable molecular dynamics^53^,^54^,^55^. However, the accelation of the guest exchange dynamics from the sufficiently slow state to the fast state may not undergo a normal peak coalescence, but display a turning point where guest H resonances start to resolve apparently owing to NMR-observable freedom of guests from cage restriction. If taking the resonance frequency separation between two slowly restricted guest signals and the turning point of guest signal shifts for Eyring analysis, the guest exchange rates and energy barriers might be estimated at 1,021, 488 s^−1^ and 55.7, 60.5 kJ mol^−1^, for *S*-BINOL⊂***Λ*****-MOC-16** and *R*-BINOL⊂***Λ*****-MOC-16** diastereomers, respectively. This means that ***Λ*****-MOC-16** can capture *S*-BINOL faster at lower energy cost than *R*-BINOL to accomplish a host–guest inclusion process.

## Discussion

**Figure 7:**
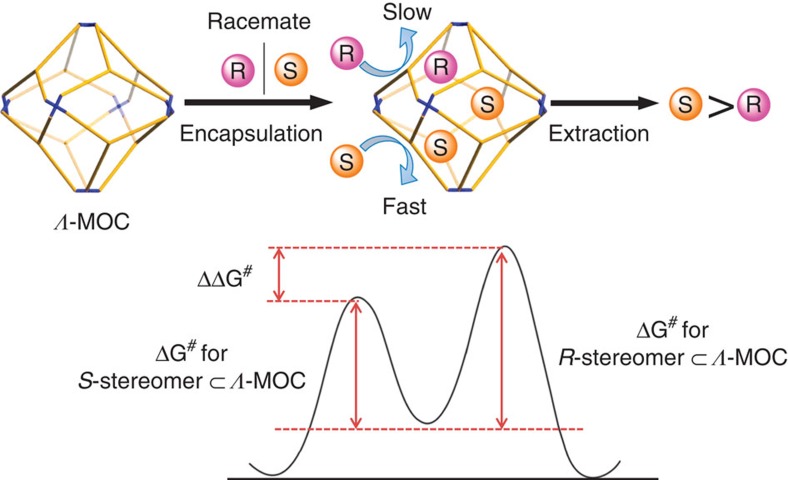
Enantioseparation mechanism. A possible resolution process relying on guest exchange dynamics of atropisomers with homochiral *Λ*-MOC-16. *Δ*G^#^ represents guest exchange energy barrier.

The host–guest dynamics and guest exchange mechanism have been vigorously explored for the insight of fundamental host–guest interactions and more complex encapsulation system design, in which both thermodynamics and kinetics play important roles in guest binding^50^,^51^,^52^,^53^,^54^,^55^. On the basis of above enantioseparation and NMR studies, we may speculate that the resolution process of homochiral ***Δ*****-** or ***Λ*****-MOCs-16** towards chiral molecules of *C*_2_ symmetry is mainly controlled by guest exchange dynamics, in comparison with the more popular thermodynamic resolution of racemic guests by chiral cages^43^. As demonstrated in Fig. 7, encapsulation of racemic *R*/*S* stereomers by, for example, ***Λ*****-MOC** may proceed in a dynamic way depending on host–guest interactions and *R*/*S*-guest competition. If inclusion of *S*-stereomers is faster than *R*-stereomers via a lower guest exchange energy barrier, preferable resolution of *S*-stereomers over *R*-stereomers is achievable. It should be noted that such a dynamic resolution based on guest exchange dynamics might be comparable but inherently different from the well-known ‘kinetic resolution' based on different reaction rates between a chiral catalyst and enantiomers^41^,^42^,^56^. Guest exchange and displacement process in a host–guest system is often sensitive to the synergistic effect from both thermodynamic and kinetic contributions^50^,^51^,^52^,^53^,^54^,^55^. Elongating resolution time may not influence *ee* results so much as by the catalytic kinetic resolution. We have tested the time-dependent chiral resolution of *R*/*S*-BINOL racemate by ***Λ*****-MOC-16**. The results indicate an increase in the *ee* value within first 2 h, but remaining nearly unchanged afterwards (Supplementary Tables 2 and 3). We believe that the distinctive host–guest dynamics between *R*-BINOL**⊂*****Λ*****-MOC-16** and *S*-BINOL**⊂*****Λ*****-MOC-16** diastereomers should originate from the stereoconfigurations of the octahedral Ru centres. The twisted arrangement of three Phen motifs around Ru centres in helical sense may not be able to differentiate inclusion behaviour of configurationally free racemic guests carrying C* stereocentres, but significantly affect the interactions between ***Δ*****-/*****Λ*****-MOCs-16** and atropisomeric guests bearing *C*_2_ symmetry owing to their intrinsic helical configurations. Formation of adaptive or mismatched host–guest diastereomers through dynamic guest exchange may be more dominated by stereochemical compatibility than by binding constant. Therefore, such stereoconfigurationally predetermined **MOCs** could afford better adaptive inclusion of one atropisomer over the opposite one, thus resulting in stereoselective separation.

In conclusion, pre-resolution of a pair of enantomeric *Δ*-/*Λ*-Ru metalloligands has been successfully implemented based on the stereogenic octahedral Ru centres in *Δ-*/*Λ*-[Ru(phen)_3_]^2+^ precursors, giving rise to the assembly of enantiopure *D*_4_-symmetric ***Δ*****-** and ***Λ*****-MOC-16** cages separately, which feature in high guest inclusion capacity and substantial stereochemical stability. The single-crystal diffraction analyses of the individual ***Δ*****-** and ***Λ*****-MOC-16** cages verified the formation of absolute ***ΔΔΔΔΔΔΔΔ***- and ***ΛΛΛΛΛΛΛΛ*** homoconfigurations, respectively, in corresponding Pd_6_(RuL_3_)_8_ cages, and the crystallization of optically pure cage products. The stereoselective inclusion of chiral molecules has been tested for two kinds of organic racemates, that is, classic chiral compounds having C* centres and atropisomeric compounds characteristic of *C*_2_ symmetry, with the phase transformation resolution processes. Successful enantioseparation of atropisomers has been accomplished by the use of these homochiral ***Δ*****-** and ***Λ*****-MOCs-16**, manifesting an unprecedented dynamic resolution process based on the kinetically driven guest exchange. The possible resolution mechanism has been investigated by the means of ^1^H NMR titration, ^1^H NMR enantiodifferentiation experiments as well as variable-temperature ^1^H NMR study. In general, this kind of assembly process may provide a new platform to study the stereochemical transmission of optically stable metal centres to versatile homochiral entities in coordination chemistry, and the dynamic resolution behaviour imposed by stereoconfiguration of metal centres might be useful in various chiral resolution of synthetic and industrial significance.

## Methods

### Materials and measurements

Unless otherwise stated, all commercial reagents and solvents were used as commercially purchased without additional purification. The NMR spectra were recorded on Bruker AVANCE III 400 (400 MHz). Circular dichroism spectra and ultraviolet–visible absorption spectra were measured with a JASCO J-810 spectropolarimeter. Specific rotations were recorded on ADP440+B+S. HR-ESI-TOF mass spectra were tested on Bruker Maxis 4G, and data analyses were processed with the Bruker Data Analysis software. HPLC spectra were measured on Agilent-2000. Diffraction data for the single crystals were collected on an Agilent SuperNova X-ray diffractometer using micro-focus dual X-ray sources (Supplementary Data 1). Syntheses and characterization details for all compounds are given in Supplementary Methods. Selected bond lengths (Å) and bond angles (°) are listed in Supplementary Tables 4–9.

### Crystal data for {*Δ*-[Pd_6_(RuL_3_)_8_](*S*-BINOL)_4_}·anion·solvent (*Δ*-MOC-16)

*I*422 space group, *a*=32.2284(4) Å, *c*=38.2801(7) Å, *V*=39,760.5(13) Å^3^, *M*r=9,727.82, *D*x=0.813 g cm^−3^, *Z*=2, *μ*=2.599 mm^−1^, 16,396 independent reflections, of which 9,021 observed (*I*>2*σ*(*I*)), *R*_1_=0.0711, *wR*_2_=0.2375, *S*=1.008, Flack parameter=0.131(13).

### Crystal data for {*Λ*-[Pd_6_(RuL_3_)_8_](*R*-BINOL)_4_}·anion·solvent (*Λ*-MOC-16)

*I*422 space group, *a*=32.5722(6) Å, *c*=38.7258(9) Å, *V*=41,086.1(19) Å^3^, *M*r=9,727.82, *D*x=0.786 g cm^−3^, *Z*=2, *μ*=2.515 mm^−1^, 17,111 independent reflections, of which 7,718 observed (*I*>2*σ*(*I*)), *R*_1_=0.0913, *wR*_2_=0.2680, *S*=1.038, Flack parameter=0.139(16).

### Crystal data for {*Δ*-[Ru(Phen)_3_](PF_6_)_2_}_2_·(C_6_H_5_CH_3_)·(CH_3_CN)_2_ (*Δ*-1)

*P*4_1_ space group, *a*=25.5619(2) Å, *c*=12.5769(2) Å, *V*=8,217.88(18) Å^3^, *M*r=2,037.48, *D*x=1.647 g cm^−3^, *Z*=4, *μ*=0.556 mm^−1^, 19,736 independent reflections, of which 17,221 observed (*I*>2*σ*(*I*)), *R*_1_=0.0678, *wR*_2_=0.1759, *S*=1.050, Flack parameter=0.00(4).

### Crystal data for {*Λ*-[Ru(Phen)_3_](PF_6_)_2_}_2_·(C_6_H_5_CH_3_)·(CH_3_CN)_2_ (*Λ*-1)

*P*4_3_ space group, *a*=25.5802(1) Å, *c*=12.5709(1) Å, *V*=8,225.73(9) Å^3^, *M*r=2,037.48, *D*x=1.645 g cm^−3^, *Z*=4, *μ*=0.556 mm^−1^, 20,142 independent reflections, of which 17,755 observed (*I*>2*σ*(*I*)), *R*_1_=0.0408, *wR*_2_=0.1071, *S*=1.025, Flack parameter=−0.033(8).

### Crystal data for *Δ*-[Ru(Phendione)_3_](ClO_4_)_2_·(H_2_O)·(CH_3_CN)_2_ (*Δ*-2)

*P*2_1_2_1_2_1_ space group, *a*=13.8114(2) Å, *b*=14.0525(2) Å, *c*=20.7957(3) Å, *V*=4,036.13(10) Å^3^, *M*r=1,028.64, *D*x=1.693 g cm^−3^, *Z*=4, *μ*=5.107 mm^−1^, 7,890 independent reflections, of which 7,536 observed (*I*>2*σ*(*I*)), *R*_1_=0.0438, *wR*_2_=0.1195, *S*=1.025, Flack parameter=−0.015(4).

### Crystal data for *Λ*-[Ru(Phendione)_3_](ClO_4_)_2_·(H_2_O)·(CH_3_CN)_2_ (*Λ*-2)

*P*2_1_2_1_2_1_ space group, *a*=13.7734(2) Å, *b*=14.0148(2) Å, *c*=20.7100(3) Å, *V*=3,997.68(10) Å^3^, *M*r=1,022.59, *D*x=1.699 g cm^−3^, *Z*=4, *μ*=5.156 mm^−1^, 7,980 independent reflections, of which 7,737 observed (*I*>2*σ*(*I*)), *R*_1_=0.0409, *wR*_2_=0.1084, *S*=1.032, Flack parameter=−0.011(3).

### General chiral resolution of racemic guests by enantiopure *Δ*/*Λ*-MOCs-16

Two kinds of methods were used to resolve racemic guests depending on whether the guest inclusion leads to precipitation. For racemic *R*/*S*-BINOL, *R*/*S*-3-Br-BINOL, *R*/*S*-6-Br-BINOL and *R*/*S*-naproxen molecules, *Method I* based on a solution–solution transfer was applied to avoid host–guest precipitation (Supplementary Fig. 25). An aqueous solution of ***Δ*****-** or ***Λ*****-MOC-16** and an ethereal solution of racemic guest were mixed and stirred vigorously at room temperature, and then the bottom layer was taken out and extracted with CHCl_3_. The extractants were combined and the solvent was removed using rotary evaporator to afford white solid as resolved guests by the homochiral MOC host. The solid was redissolved in isopropanol and the *ee* of guest molecules was determined using HPLC. For racemic *R*/*S*-spirodiol, *R*/*S*-1-(1-naphthyl)ethanol and *R*/*S*-benzoin molecules, Method II based on a solid-solution transfer was applied directly. The powder of guest racemate was suspended in the aqueous solution of ***Δ-***or ***Λ*****-MOC-16**. The mixture was stirred vigorously at room temperature. After centrifugation, the filtrate was collected and extracted with CHCl_3_. The extractants were combined and the solvent was removed by rotary evaporator to afford white solid as resolved guest. The *ee* analysis is the same as in Method I.

## Additional information

**Accession codes:** The X-ray crystallographic coordinates for structures reported in this Article have been deposited at the Cambridge Crystallographic Data Centre (CCDC), under deposition number CCDC 1432349–1432354. These data can be obtained free of charge from The Cambridge Crystallographic Data Centre via www.ccdc.cam.ac.uk/data_request/cif.

**How to cite this article:** Wu, K. *et al.* Homochiral *D*_4_-symmetric metal–organic cages from stereogenic Ru(II) metalloligands for effective enantioseparation of atropisomeric molecules. *Nat. Commun.* 7:10487 doi: 10.1038/ncomms10487 (2016).

## Supplementary Material

Supplementary Information Supplementary Figures 1-29, Supplementary Tables 1-9 and Supplementary Methods

Supplementary Data 1 Crystallographic Information Files

## Figures and Tables

**Table 1:**
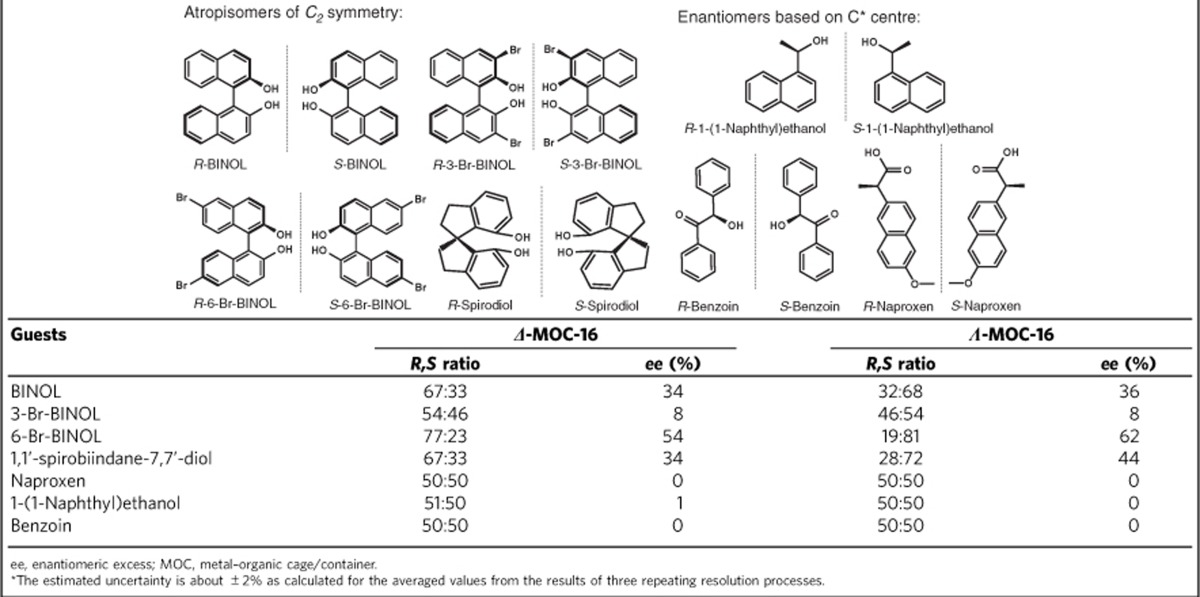
Enantioselective resolution of chiral organic molecules*.
