# Supplementary material for: Homochiral D4-symmetric metal–organic cages from stereogenic Ru(II) metalloligands for effective enantioseparation of atropisomeric molecules
Source: Nat Commun. 2016 Feb 3;7:10487. doi: 10.1038/ncomms10487 (PMC4742817; doi:10.1038/ncomms10487)
Supplement: Supplementary Information — Supplementary Figures 1-29, Supplementary Tables 1-9 and Supplementary Methods [file ncomms10487-s1.pdf]

Supplementary Information

Supplementary Figures

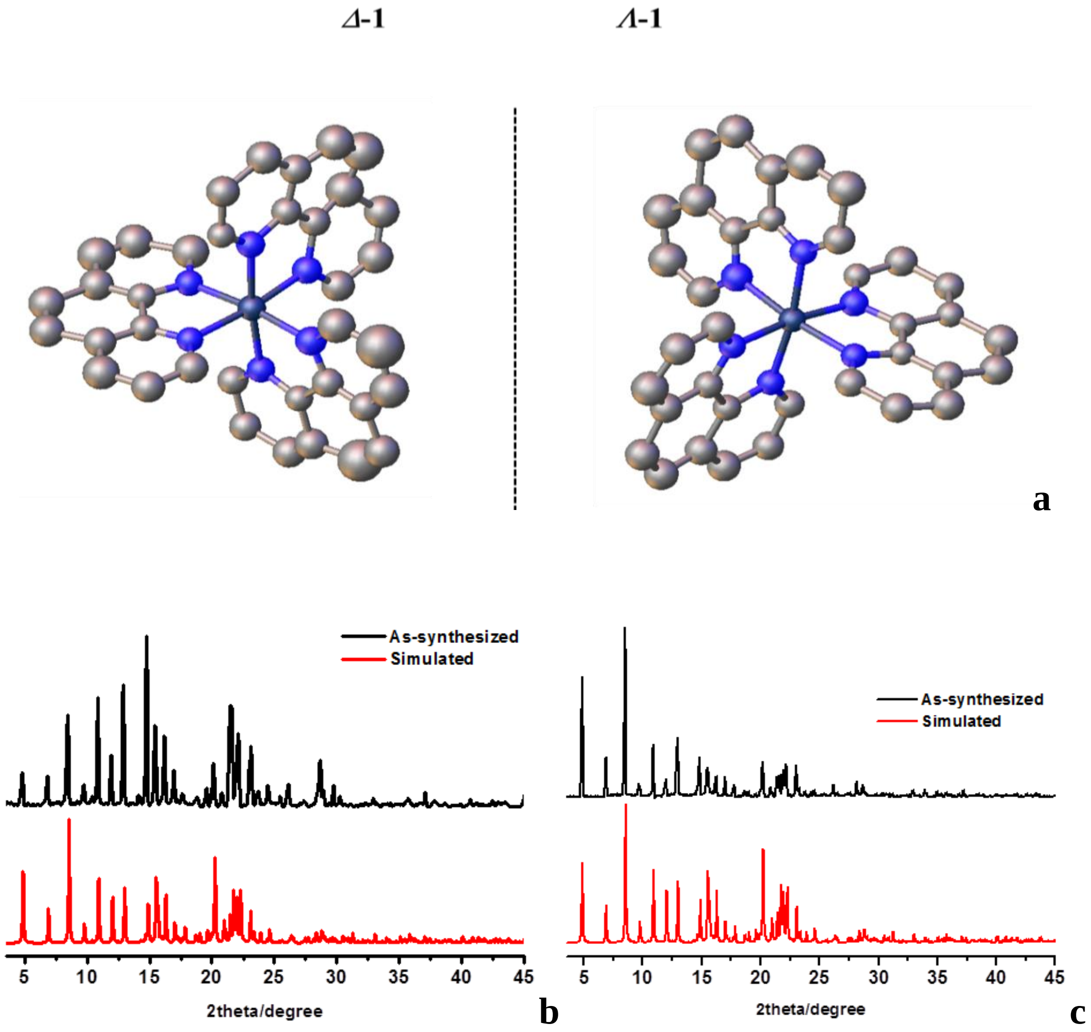

Supplementary Figure 1 | X-Ray single-crystal structures and powder diffraction. (a) A pair of enantiomeric $[Ru(Phen)_3]^{2+}$ motifs in ***Δ*****-** and ***Λ*****-1-PF**$_6$ (solvents, anions and H atoms are omitted for clarity). **(b,c)** Powder diffraction patterns of as-synthesized sample and single-crystal simulation for ***Δ*****--1-PF**$_6$ and ***Λ*****-1-PF**$_6$, respectively, confirming the phase purity of bulk sample.

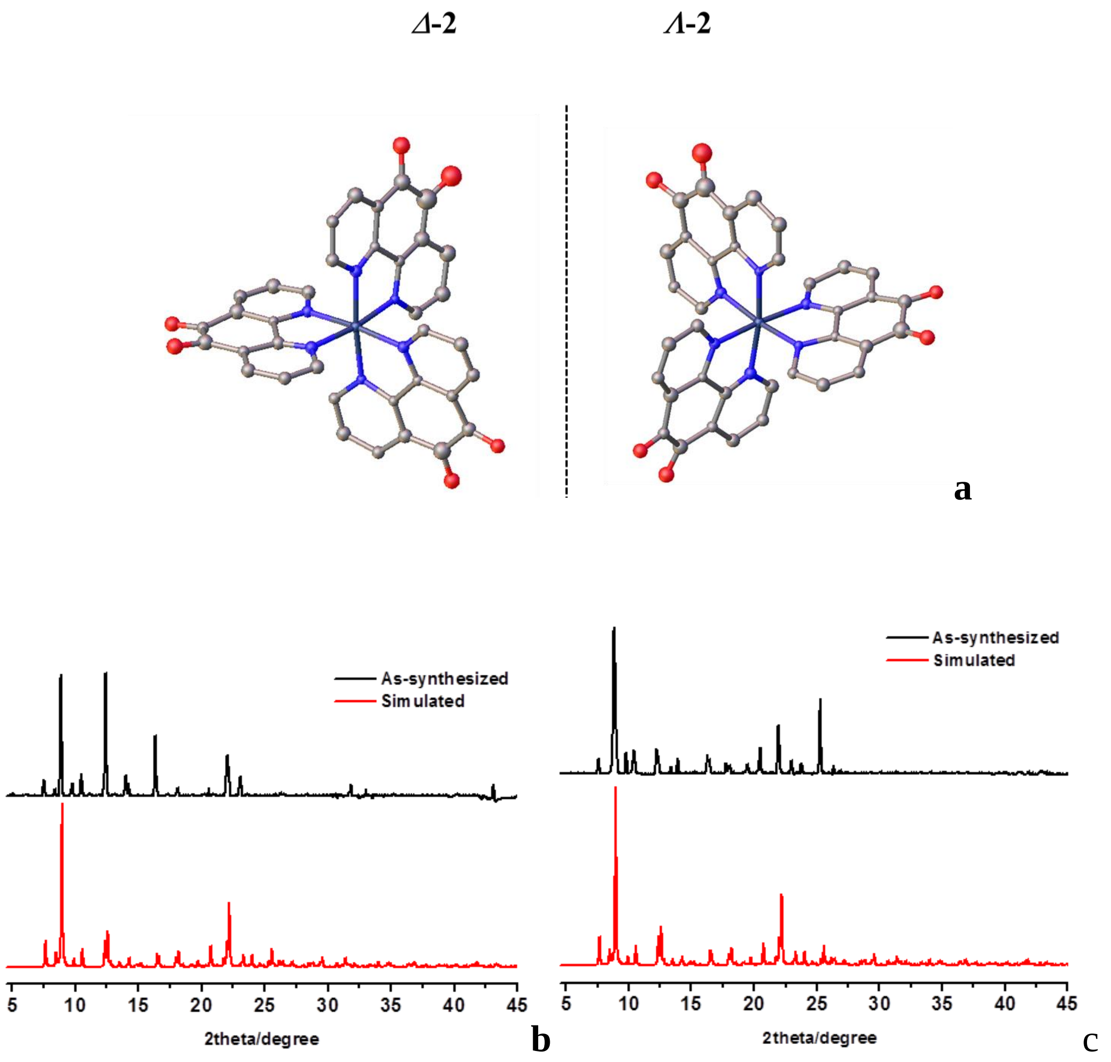

Supplementary Figure 2 | X-Ray single-crystal structures and powder diffraction. (a) A pair of enantiomeric [Ru(Phendione)$_3$]$^{2+}$ motifs in ***Δ*-** and ***Λ*-2-ClO$_4$** (solvents, anions and H atoms are omitted for clarity). **(b,c)** Powder diffraction patterns of as-synthesized sample and single-crystal simulation for ***Δ*-2-ClO$_4$** and ***Λ*-2-ClO$_4$**, respectively, confirming the phase purity of bulk sample.

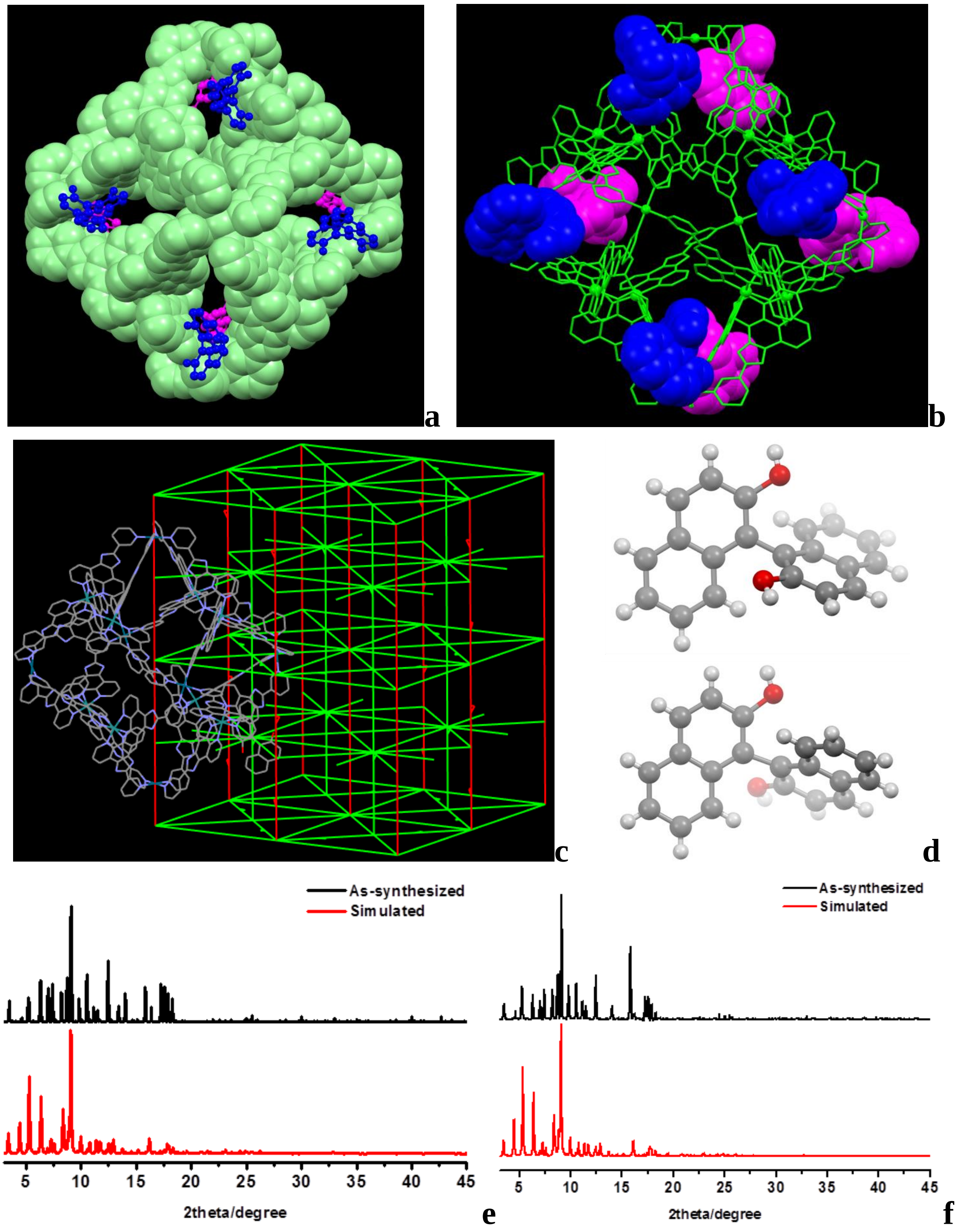

Supplementary Figure 3 | Crystal structures. a) A **Δ-MOC-16** cage (in space-filling mode) capturing eight *S*-BINOL guests (in ball-and-stick mode) onto the windows pockets. **b)** A **Λ-MOC-16** cage (in ball-and-stick mode) capturing eight *R*-BINOL guests (in space-filling mode) onto the windows pockets. **c)** Symmetry elements in D_4-symmetric **Δ-MOCs-16** crystal lattice showing positions of C_4 (red lines) and C_2 (green lines). **d)** Molecular structures of *S*-BINOL and *R*-BINOL guests. **e,f)** Powder diffraction patterns of as-synthesized sample and single-crystal simulation for **Δ-MOC-16** and **Λ-MOC-16**, respectively, confirming the phase purity of bulk sample.

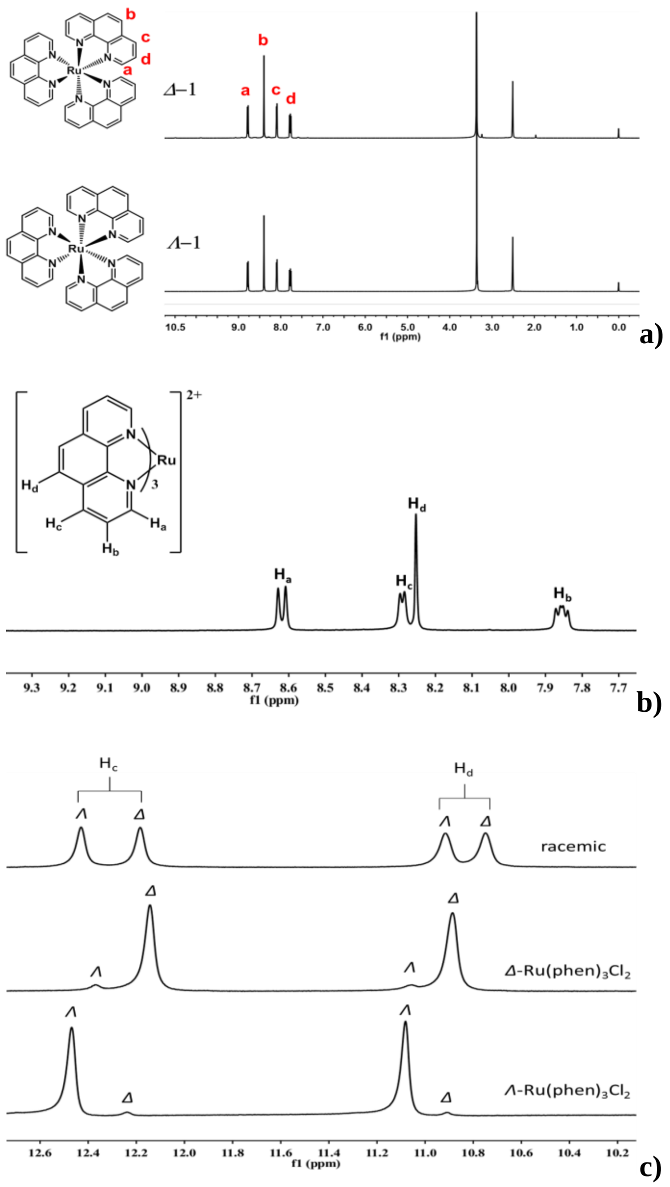

Supplementary Figure 4 | a) ^{1}H NMR spectra of ***Δ*/*Λ*-1-PF$_6$** in DMSO-d_6. **b)** ^{1}H NMR spectrum of ***Δ*-[Ru(phen)$_3$]Cl$_2$ (*Δ*-1-Cl)** in CD_2Cl_2. Proton assignment shown in the inset. **c)** ^{1}H NMR spectra of the racemic and enantiomeric **[Ru(phen)$_3$]Cl$_2$** (10 mM) in the presence of Eu((+)tfc)$_3$ (20 mM) in CD_2Cl_2. Only the region around the H_c and H_d protons are shown.

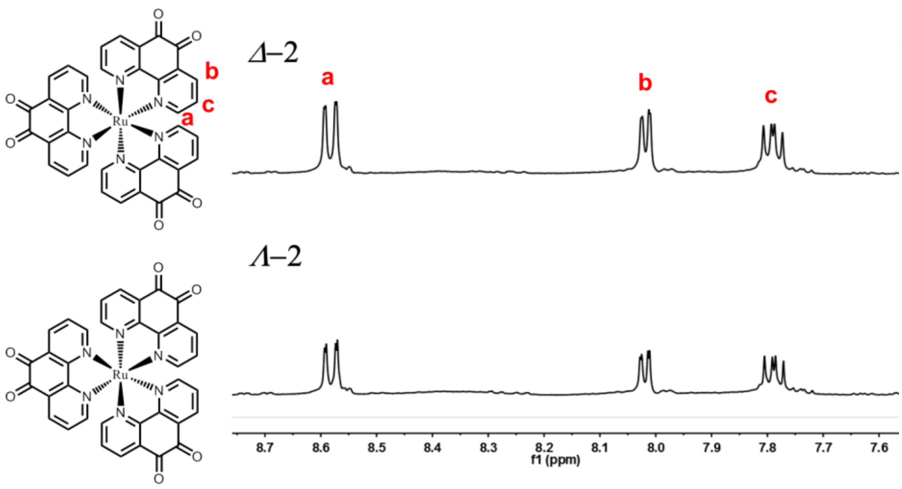

Supplementary Figure 5 | ^{1}H NMR spectra of ***Δ-/Λ*-2-PF$_6$** in DMSO-d_6.

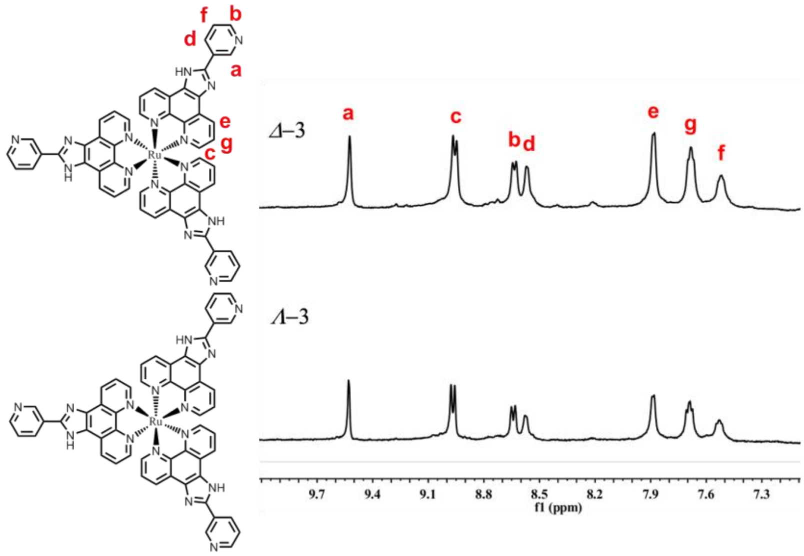

Supplementary Figure 6 | ^{1}H NMR spectra of *Δ*-/*Λ*-3-PF$_6$ in DMSO-*d*$_6$.

$[\mathit{\Delta}\text{-Ru(L)}_3]^{2+}$

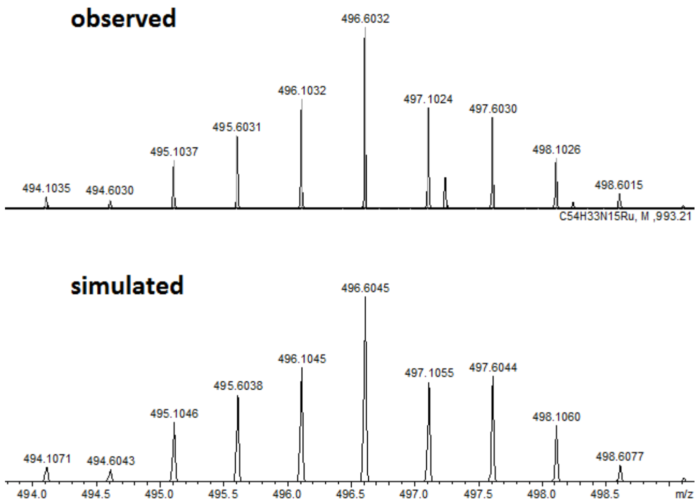

$[\mathit{\Delta}\text{-Ru(L)}_3\text{-H}^+]^+$

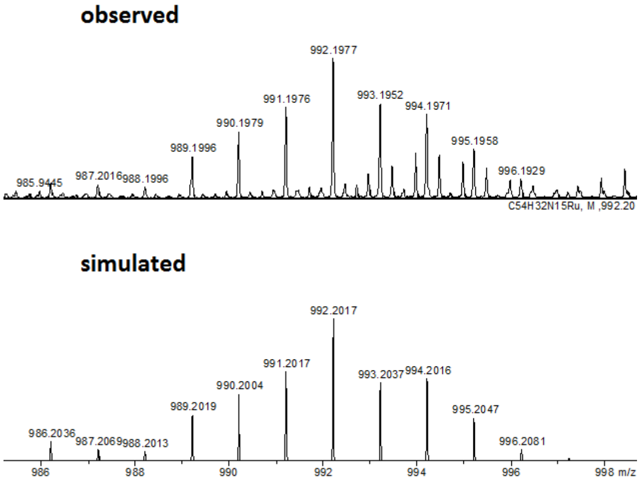

$[\mathit{\Lambda}\text{-Ru(L)}_3]^{2+}$

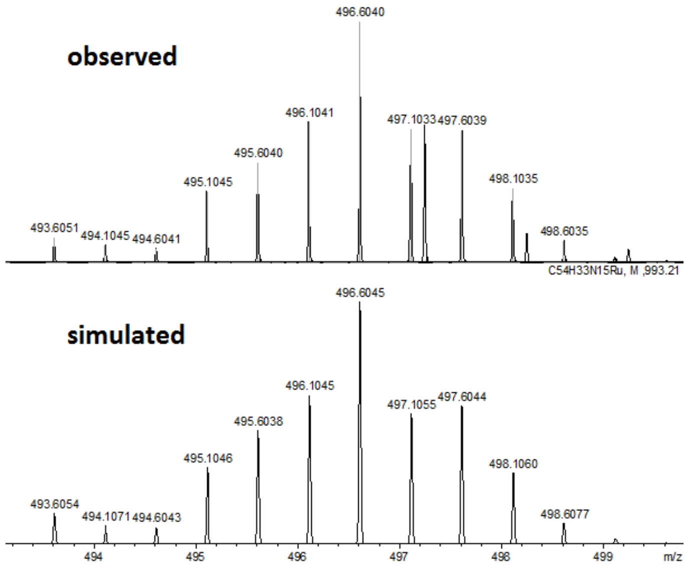

[Λ-Ru(L)$_3$-H$^+$]$^+$

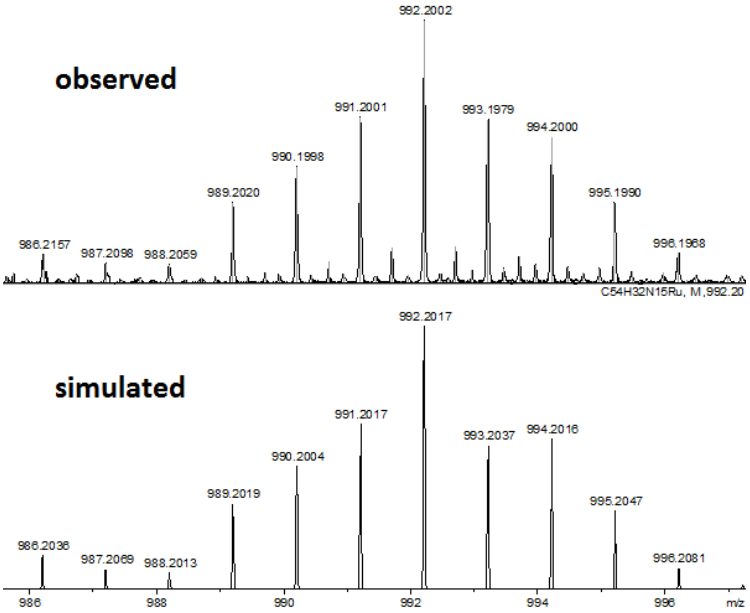

Supplementary Figure 7 │ HRESI-TOF-MS spectra of *Δ*-/*Λ*-3-PF$_6$ in DMSO-CH$_3$CN (v:v = 1:20).

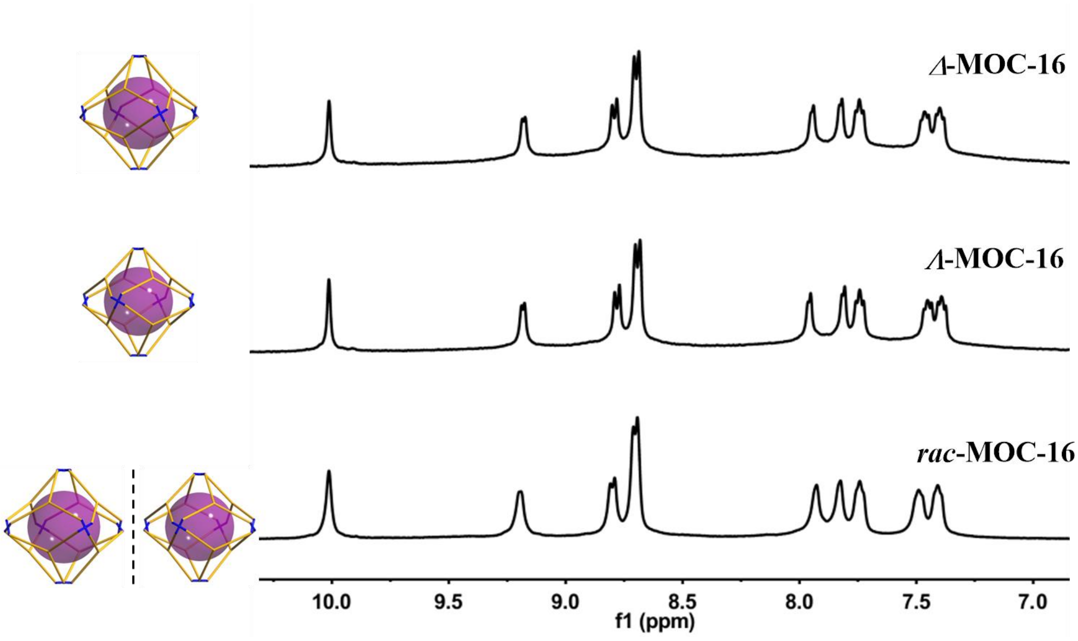

a

Supplementary Figure 8 | ^{1}H NMR comparison of the racemic and enantiomeric $Pd_6(RuL_3)_8$ MOCs in DMSO-*d*$_6$-D_2O (v:v = 1:5) solution.

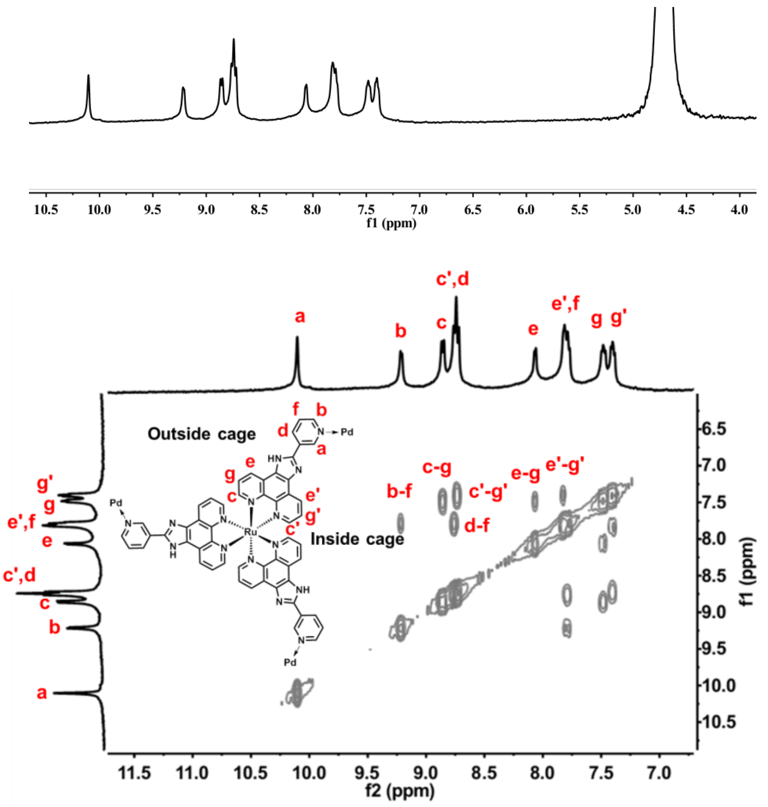

a

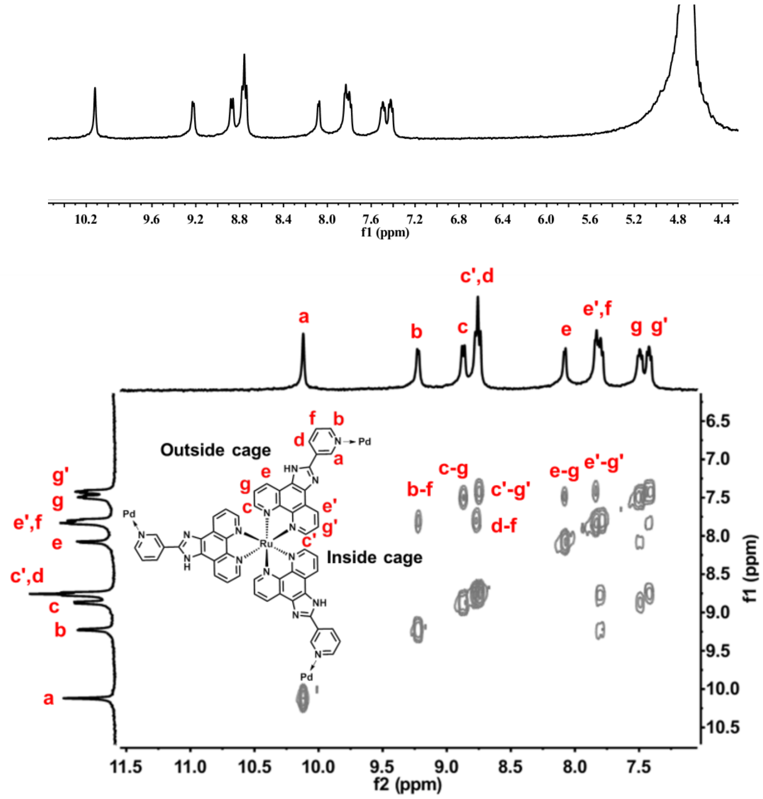

b

Supplementary Figure 9 | a) ^{1}H NMR and ^{1}H-^{1}H-COSY spectra of ***Δ*-MOC-16** in D_2O. **b**) ^{1}H NMR and ^{1}H-^{1}H-COSY spectra of ***Λ*-MOC-16** in D_2O.

9^+: $[(Pd_6(RuL_3)_8)\text{-}19H^+]^{9+}$

Selected mass information in the 9^+ peak ($[(Pd_6(RuL_3)_8)\text{-}19H^+]^{9+}$)						
**Observed Ion (m/z)**	**Intensity**	**Resolution**	**FWHM**	**Theoretical mass**	**Difference (obs.-theo.)**	**Error (ppm)**
951.1081	229	30286	0.0314	951.1068	0.0013	1.37
951.2146	237	36481	0.0261	951.2179	-0.0033	-3.5
951.3327	237	27437	0.0347	951.3290	0.0037	3. 9
951.4377	243	34025	0.0280	951.4401	-0.0024	-2.5
951.5542	251	29289	0.0325	951.5513	0.0029	3.1
951.6611	285	43737	0.0218	951.6624	-0.0013	-1.4
951.7644	278	28315	0.0336	951.7735	-0.0091	-9.6

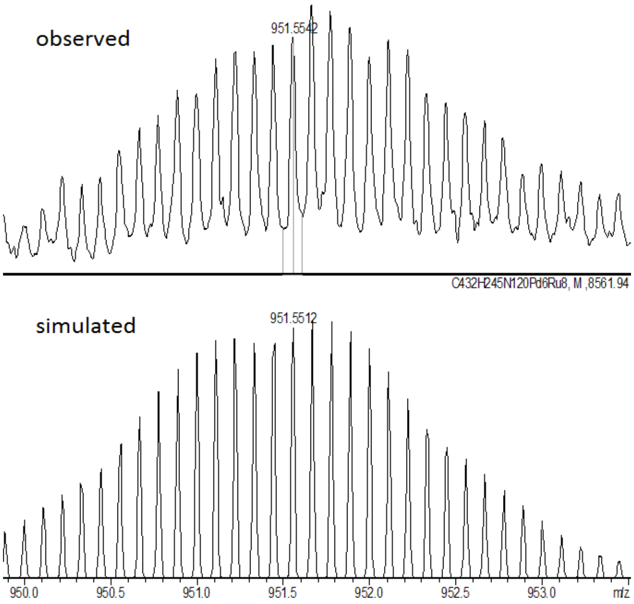

8^+: $[(Pd_6(RuL_3)_8)\text{-}20H^+]^{8+}$

Selected mass information in the 8^+ peak ($[(Pd_6(RuL_3)_8)\text{-}20H^+]^{8+}$)						
**Observed Ion (m/z)**	**Intensit y**	**Resolution**	**FWHM**	**Theoretical mass**	**Difference (obs.-theo.)**	**Error (ppm)**
1070.1168	1150	28144	0.0380	1070.1192	-0.0024	-2.2
1070.2425	1112	26729	0.0400	1070.2443	-0.0018	-1.7
1070.3677	1212	26948	0.0397	1070.3693	-0.0016	-1.5
1070.4916	1132	29819	0.0359	1070.4943	-0.0027	-2.5
1070.6180	1149	29396	0.0364	1070.6193	-0.0013	-1.2
1070.7419	1105	23278	0.0364	1070.7443	-0.0024	-2.2
1070.8675	1083	29293	0.0460	1070.8694	-0.0019	-1.8

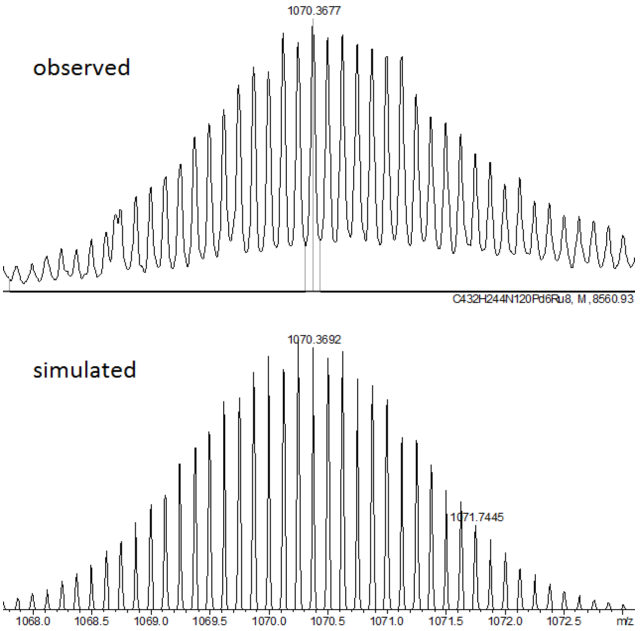

7^{+}: $[(Pd_6(RuL_3)_8)\text{-}21H^{+}]^{7+}$

Selected mass information in the 9^{+} peak ($[(Pd_6(RuL_3)_8)\text{-}21H^{+}]^{7+}$)						
**Observed Ion (m/z)**	**Intensity**	**Resolution**	**FWHM**	**Theoretical mass**	**Difference (obs.-theo.)**	**Error (ppm)**
1223.1338	1094	23904	0.0512	1223.1353	-0.0015	1.2
1223.2782	1128	39307	0.0311	1223.2781	0.0001	0.1
1223.4162	1313	28789	0.0425	1223.4210	-0.0048	-3.9
1223.5607	1150	38288	0.0320	1223.5639	-0.0032	-2.6
1223.7056	1163	25324	0.0483	1223.7068	-0.0012	-1.0
1223.8452	1058	43532	0.0281	1223.8497	-0.0045	-3.7
1223.9889	1079	37691	0.0325	1223.9926	-0.0037	-3.0

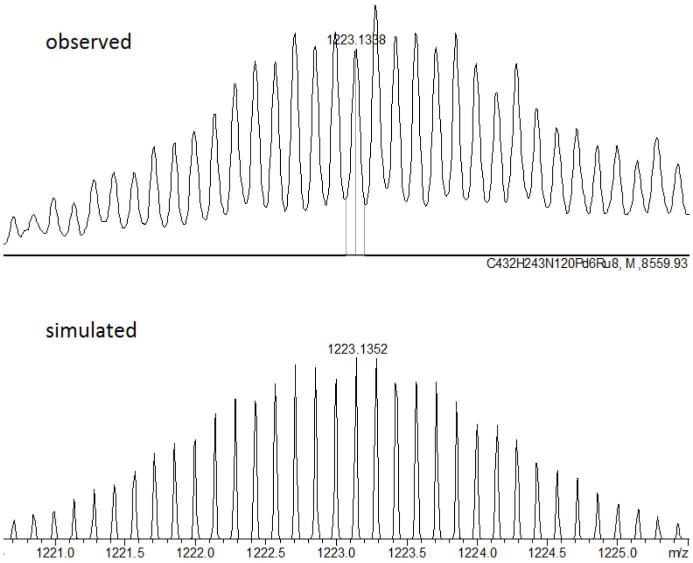

Supplementary Figure 10 | HRESI-TOF-MS spectra of *Λ*-MOC-16 species with different valence states in DMSO-CH_3CN (v:v=1:20) solution.

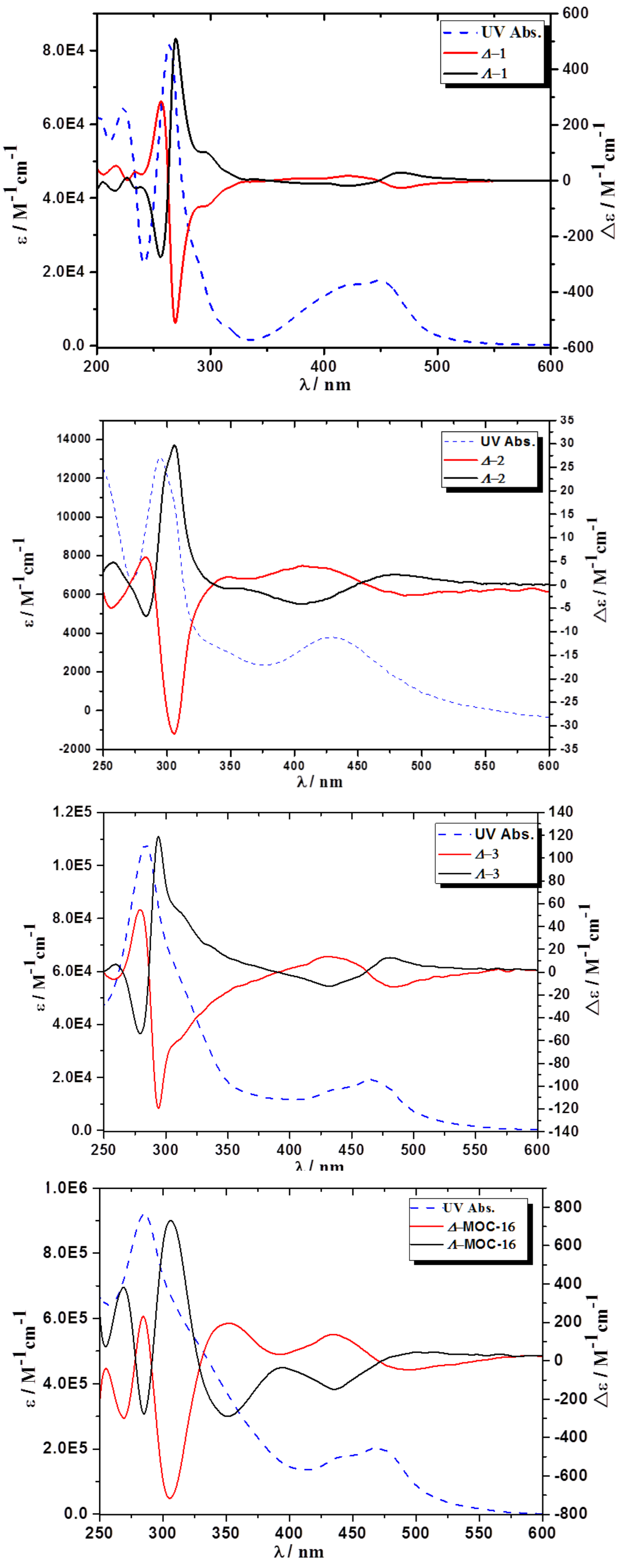

Supplementary Figure 11 | From top to bottom: CD (solid lines) and UV (dotted lines) spectra of *Δ*-/*Λ*-1 (in MeCN), *Δ*-/*Λ*-2 (in H_2O), *Δ* -/*Λ*-3 (in DMSO) and *Δ* -/*Λ*-MOCs-16 (in H_2O).

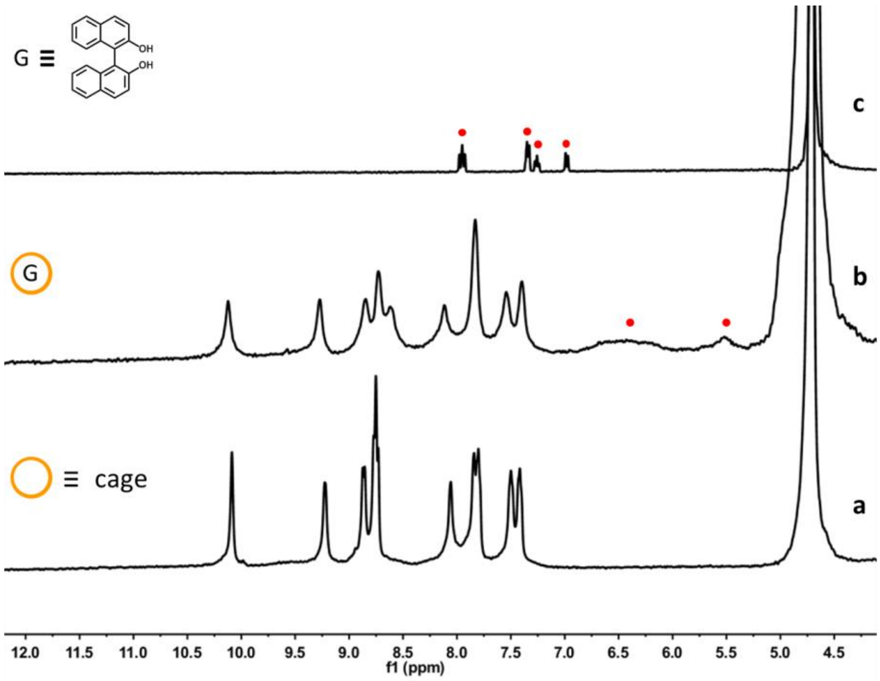

Supplementary Figure 12 | ^{1}H NMR study of guest inclusion by racemic MOC-16: (a) free cage, **(b)** BINOL⊂cage at RT in D_2O, and **(c)** free BINOL in DMSO-d_6/D_2O (v:v = 1:2). Signals of guests show in red balls.

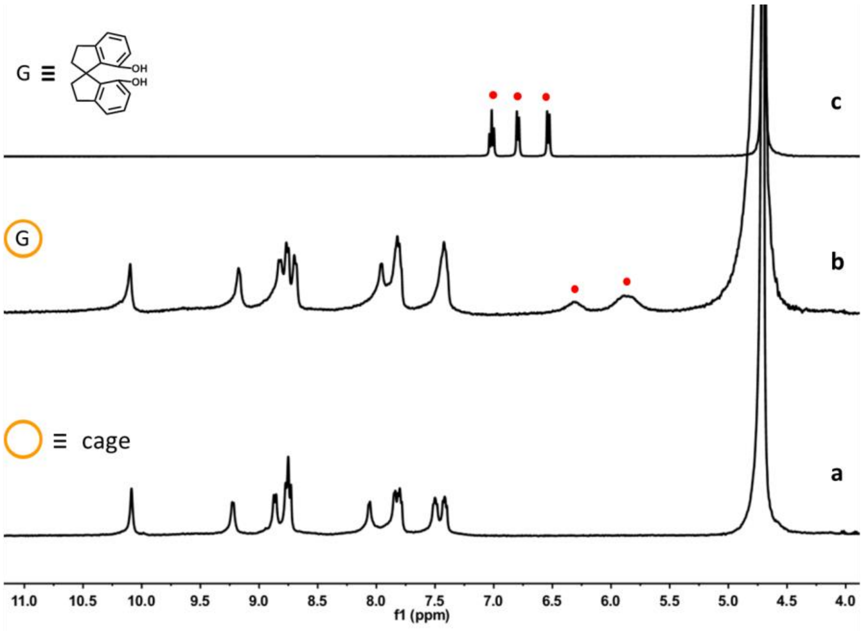

Supplementary Figure 13 | ^{1}H NMR study of guest inclusion by racemic MOC-16: (a) free cage, **(b)** spirodiol ⊂cage at RT in D_2O, and **(c)** free spirodiol in DMSO-d_6/D_2O (v:v=1:2). Signals of guests show in red balls.

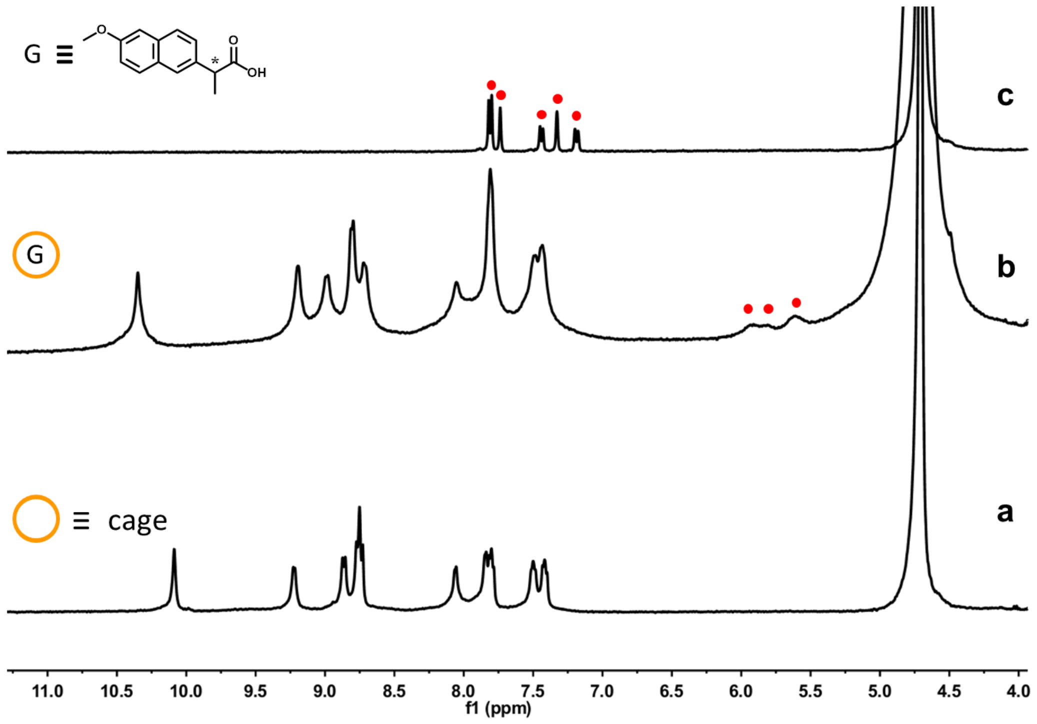

Supplementary Figure 14 | ^{1}H NMR study of guest inclusion by racemic MOC-16: (a) free cage, **(b)** naproxen⊂cage at RT in D_2O, and **(c)** free naproxen in DMSO-d_6/D_2O (v:v=1:2). Signals of guests show in red balls.

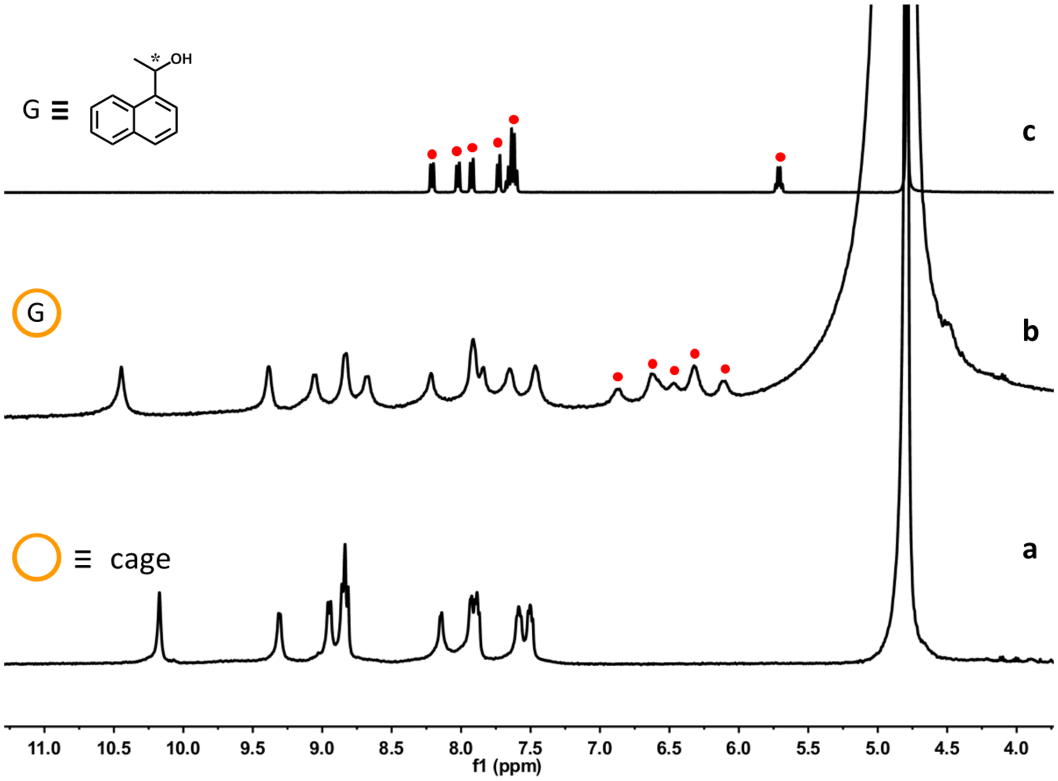

Supplementary Figure 15 | ^{1}H NMR study of guest inclusion by racemic MOC-16: (a) free cage, **(b)** 1-(1-Naphthyl)ethanol⊂cage at RT in D_2O, and **(c)** free 1-(1-Naphthyl)ethanol in DMSO-d_6/D_2O (v:v=1:2). Signals of guests show in red balls.

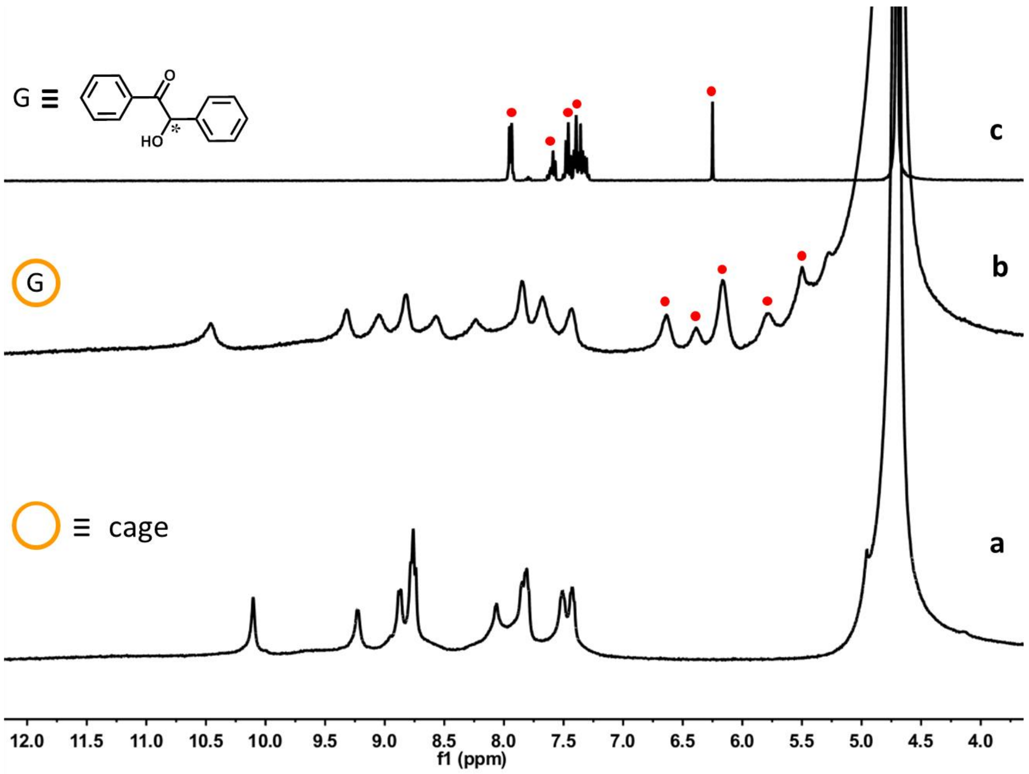

Supplementary Figure 16 | ^{1}H NMR study of guest inclusion by racemic MOC-16: (a) free cage, **(b)** benzoin⊂cage at RT in D_2O, and **(c)** free benzoin in DMSO-d_6/D_2O (v:v=1:2). Signals of guests show in red balls.

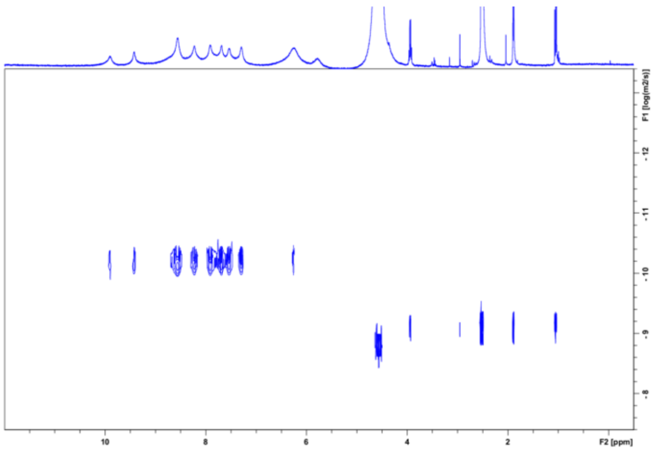

Supplementary Figure 17 | DOSY spectrum of the *R*-BINOL ⊂ *Λ*-MOC-16 system in a mixture of DMSO-d_6/D_2O = 1/5.

Δ-MOC-16:

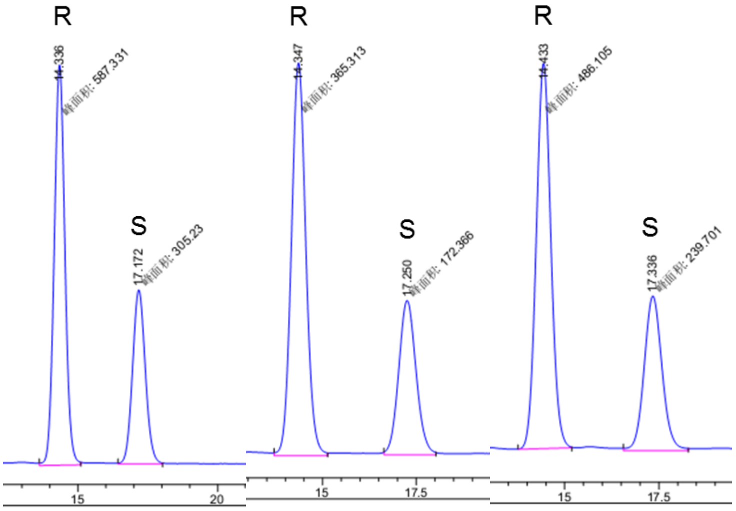

	area(%)		area(%)		area(%)
R	68	*R*	66	*R*	67
S	32	*S*	34	*S*	33
ee.	36(*R*)	ee.	32(*R*)	ee.	34(*R*)

Λ-MOC-16:

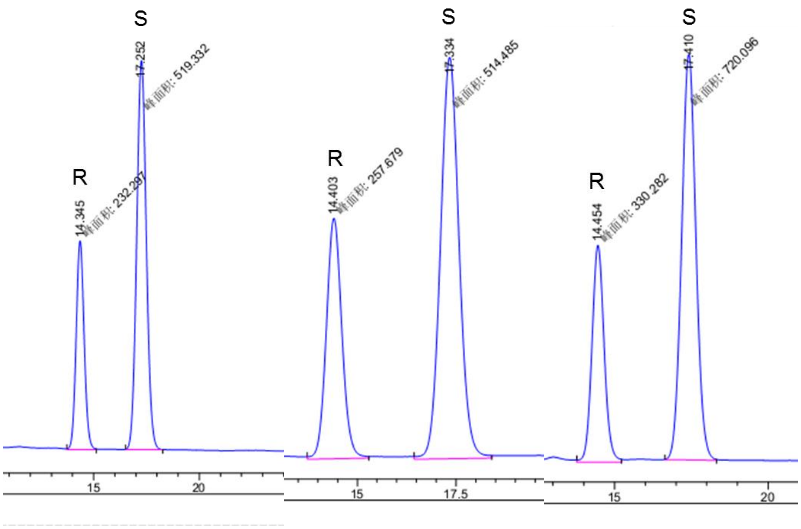

	area(%)		area(%)		area(%)
R	31	*R*	33	*R*	31
S	69	*S*	67	*S*	68
ee.	38(*S*)	ee.	34(*S*)	ee.	37(*S*)

Supplementary Figure 18 | Chiral resolution result of (±)-1,1'-Bi(2-naphthol) (BINOL).

Δ-MOC-16:

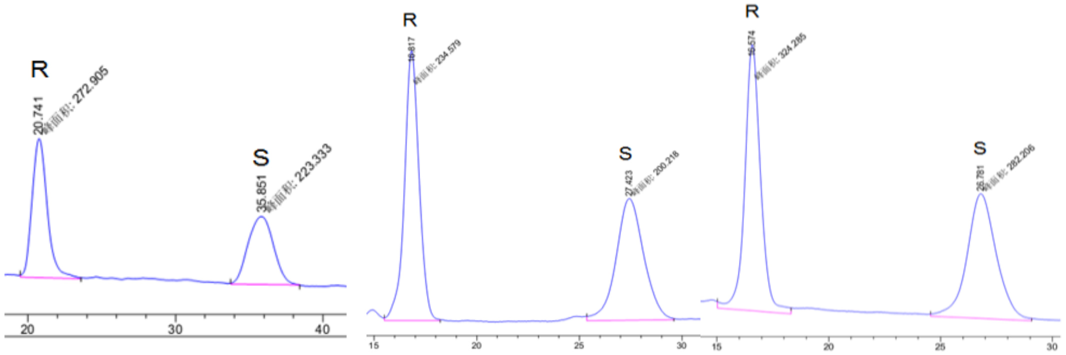

	area(%)		area(%)		area(%)
R	55	*R*	54	*R*	53.5
S	45	*S*	46	*S*	46.5
ee.	10(*R*)	ee.	8(*R*)	ee.	7(*R*)

Λ-MOC-16:

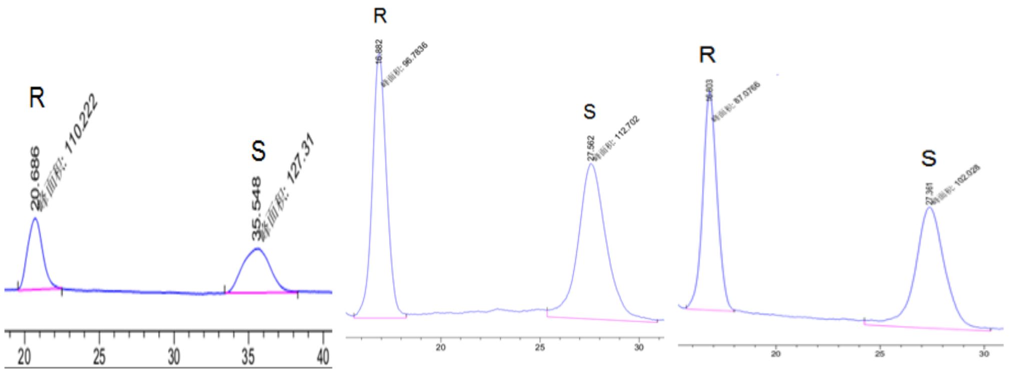

	area(%)		area(%)		area(%)
R	46.5	*R*	46	*R*	46
S	53.5	*S*	54	*S*	54
ee.	7(*S*)	ee.	8(*S*)	ee.	8(*S*)

Supplementary Figure 19 | Chiral resolution result of (±)-3,3'-Dibromo-1,1'-bi-2-naphthol (3-Br-BINOL).

Δ-MOC-16:

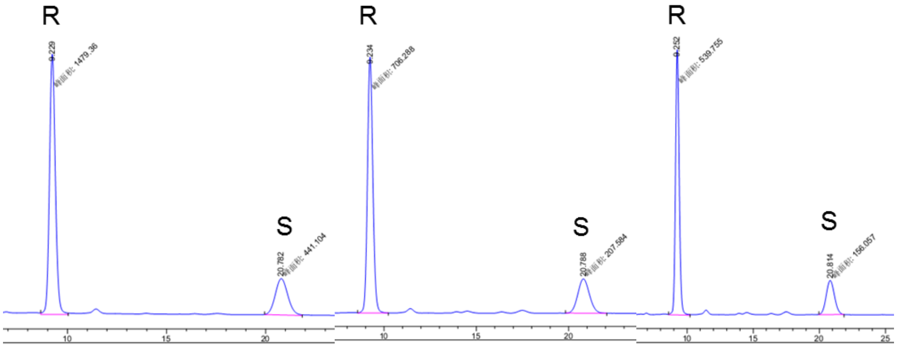

	area(%)		area(%)		area(%)
R	77	*R*	77	*R*	77.5
S	23	*S*	23	*S*	22.5
ee.	54(*R*)	ee.	54(*R*)	ee.	55 (*R*)

Λ-MOC-16:

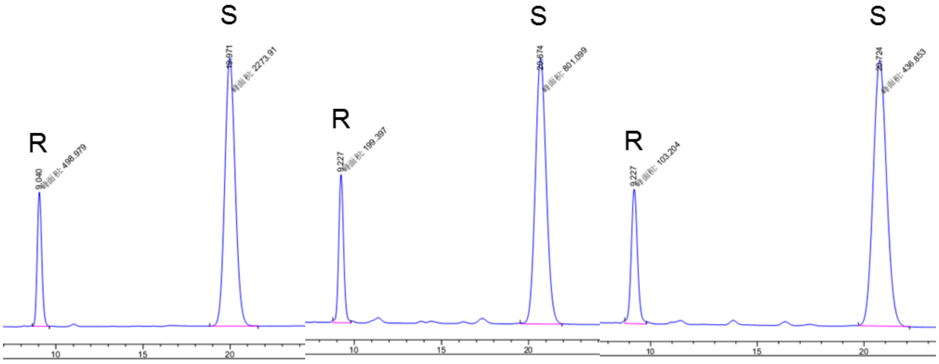

	area(%)		area(%)		area(%)
R	18	*R*	20	(*R*)	*R*
S	82	*S*	80	(*S*)	*S*
ee.	64(*S*)	ee.	60(*S*)	ee.	62(*S*)

Supplementary Figure 20 | Chiral resolution result of (±)-6,6'-Dibromo-1,1'-bi-2-naphthol (6-Br-BINOL).

Δ-MOC-16:

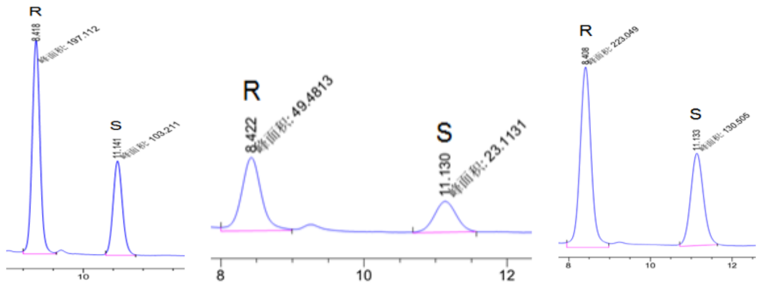

	area(%)		area(%)		area(%)
R	66	*R*	68	*R*	67
S	34	*S*	32	*S*	33
ee.	32(*R*)	ee.	36(*R*)	ee.	34(*R*)

Λ-MOC-16:

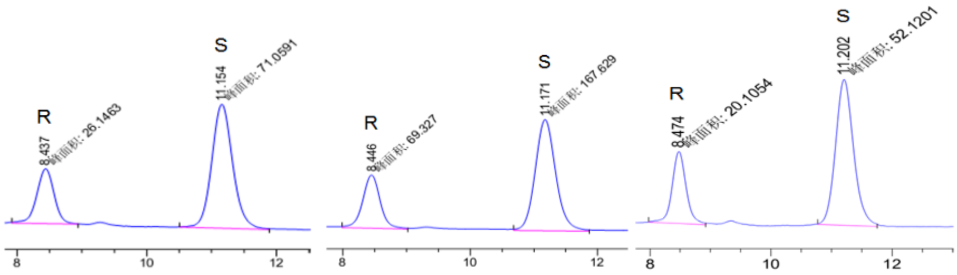

	area(%)		area(%)		area(%)
R	27	*R*	29	*R*	28
S	73	*S*	71	*S*	72
ee.	46(*S*)	ee.	42(*S*)	ee.	44(*S*)

Supplementary Figure 21 | Chiral resolution result of (±)-1,1'-spirobiindane-7,7'-diol (Spirodiol).

Δ-MOC-16:

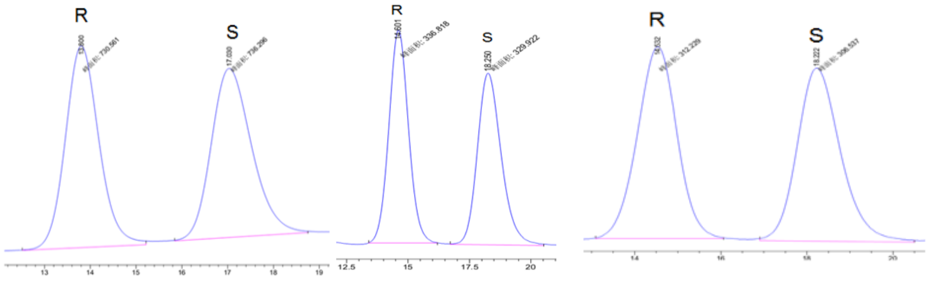

	area(%)		area(%)		area(%)
R	50	*R*	50.5	*R*	50.5
S	50	*S*	49.5	*S*	49.5
ee.	0	ee.	1(*R*)	ee.	1(*R*)

Λ-MOC-16:

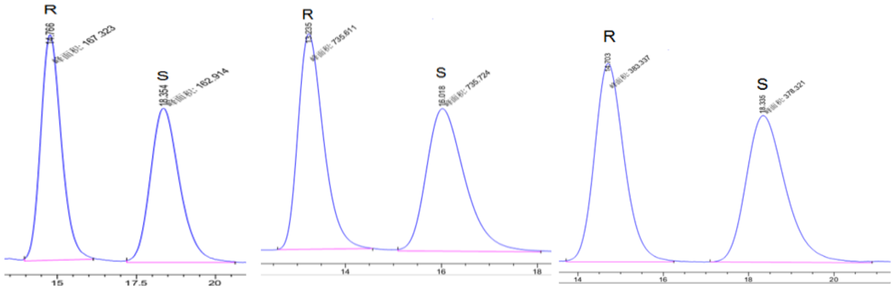

	area(%)		area(%)		area(%)
R	51	*R*	50	*R*	50
S	49	*S*	50	*S*	50
ee.	2(*R*)	ee.	0	ee.	0

Supplementary Figure 22 | Chiral resolution result of (±)-2-(6-Methoxynaphthalen -2-yl)propanoic acid (Naproxen).

***Δ*-MOC-16:**

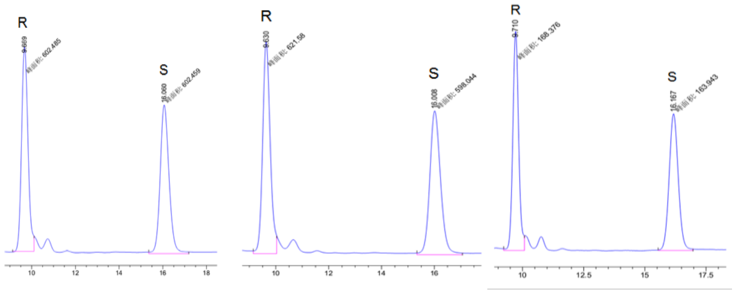

	area(%)		area(%)		area(%)
R	50	*R*	51	*R*	51
S	50	*S*	49	*S*	49
ee.	0	ee.	2(*R*)	ee.	2(*R*)

***Λ*-MOC-16:**

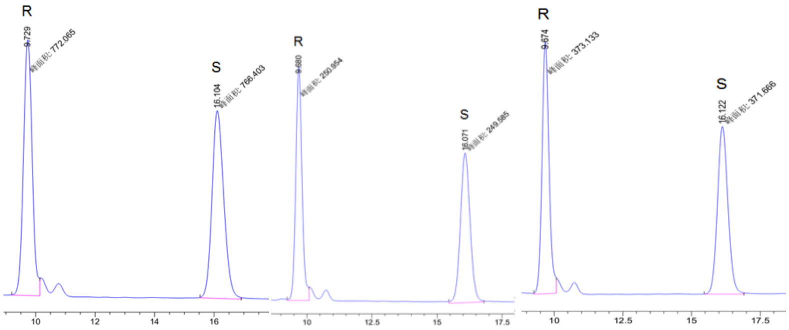

	area(%)		area(%)		area(%)
R	50	*R*	50	*R*	50
S	50	*S*	50	*S*	50
ee.	0	ee.	0	ee.	0

Supplementary Figure 23 | Chiral resolution result of (±)-1-(1-naphthyl)ethanol.

Δ-MOC-16:

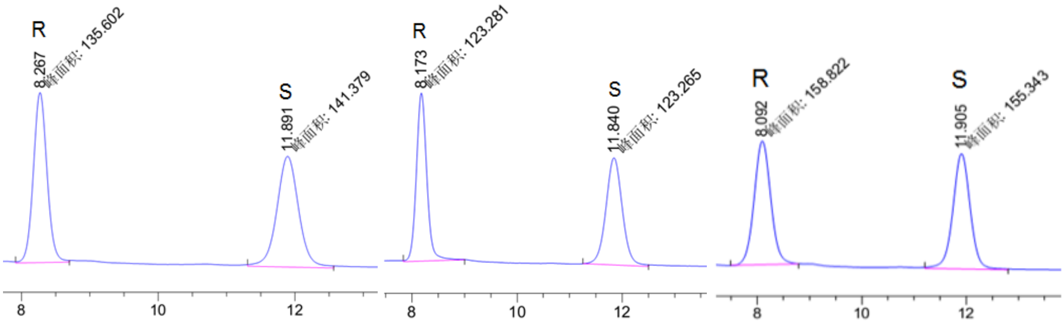

	area(%)		area(%)		area(%)
R	49	*R*	50	*R*	50.5
S	51	*S*	50	*S*	49.5
ee.	2(*S*)	ee.	0	ee.	1(*R*)

Λ-MOC-16:

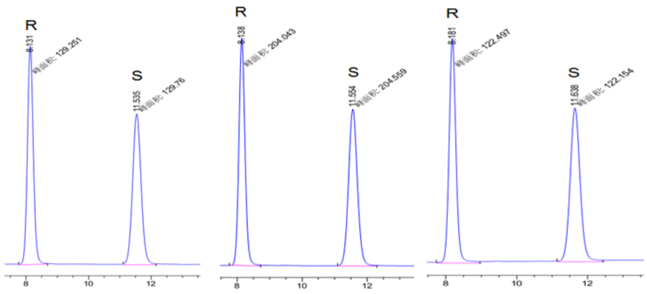

	area(%)		area(%)		area(%)
R	50	*R*	50	*R*	50
S	50	*S*	50	*S*	50
ee.	0	ee.	0	ee.	0

Supplementary Figure 24 | Chiral resolution result of (±)-2-Hydroxy-2-phenyl acetophenone.

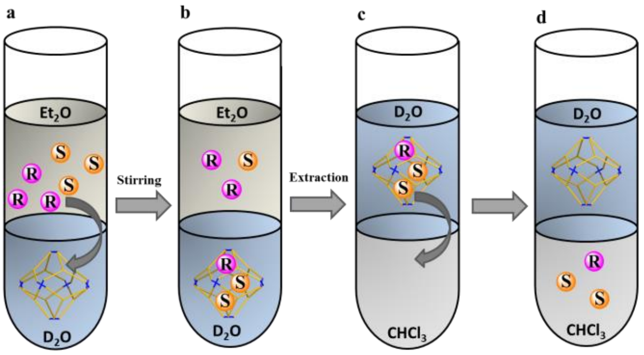

Supplementary Figure 25 | Schematic representation of chiral resolution process based on a solution-solution transfer method. (**a**) Two separate solution layers of Et_2O dissolving racemic guests and D_2O dissolving ***Λ*-MOC-16**. (**b**) Transformation of different amount of *R*- and *S*-stereomers from Et_2O phase to D_2O phase through inclusion with ***Λ*-MOC-16.** (**c**) Extraction of encapsulated guests from ***Λ*-MOC-16** by $CHCl_3$. (**d**) Separation of chiral resolution product and recovery of empty ***Λ*-MOC-16**.

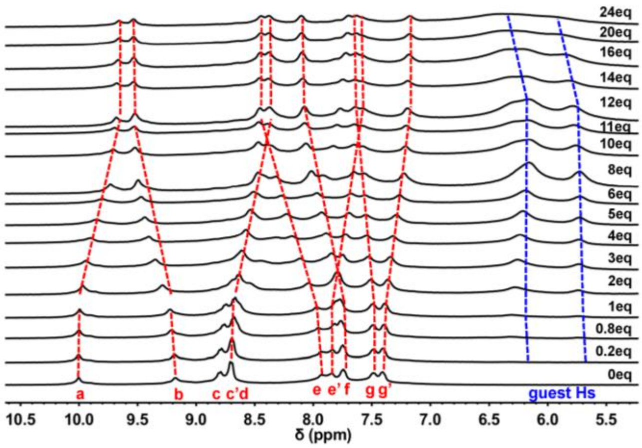

a

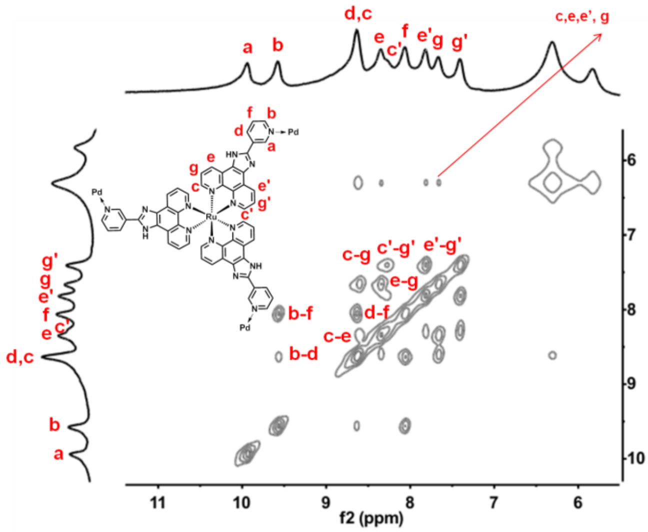

b

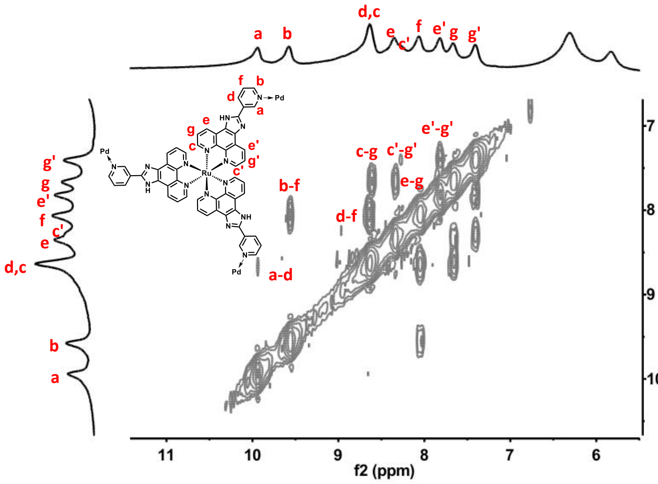

c

Supplementary Figure 26 | (a) ^{1}H NMR titration of ***R*-BINOL** into ***Λ*-MOC-16** (DMSO-d_6/D_2O = 1/5, 298 K). Red lines show host protons while blue lines show guest protons. **(b)** NOESY of ***R*-BINOL ⊂ *Λ*-MOC-16** in a mixture of DMSO-d_6/D_2O = 1/5. **(c)** ^{1}H-^{1}H-COSY of ***R*-BINOL ⊂ *Λ*-MOC-16** in a mixture of DMSO-d_6/D_2O = 1/5.

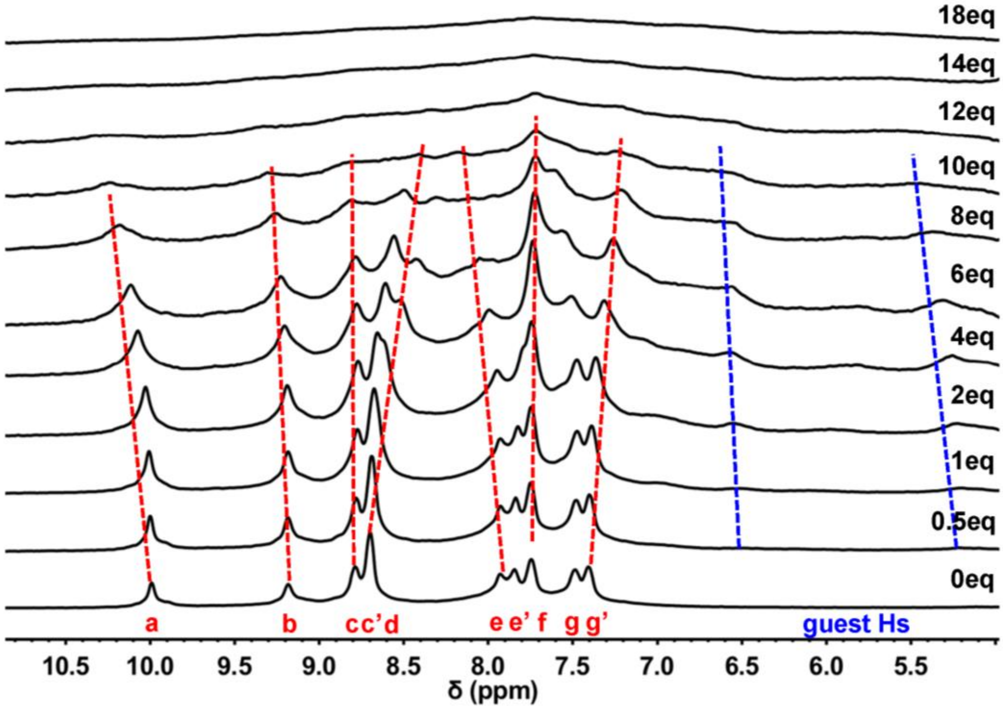

a

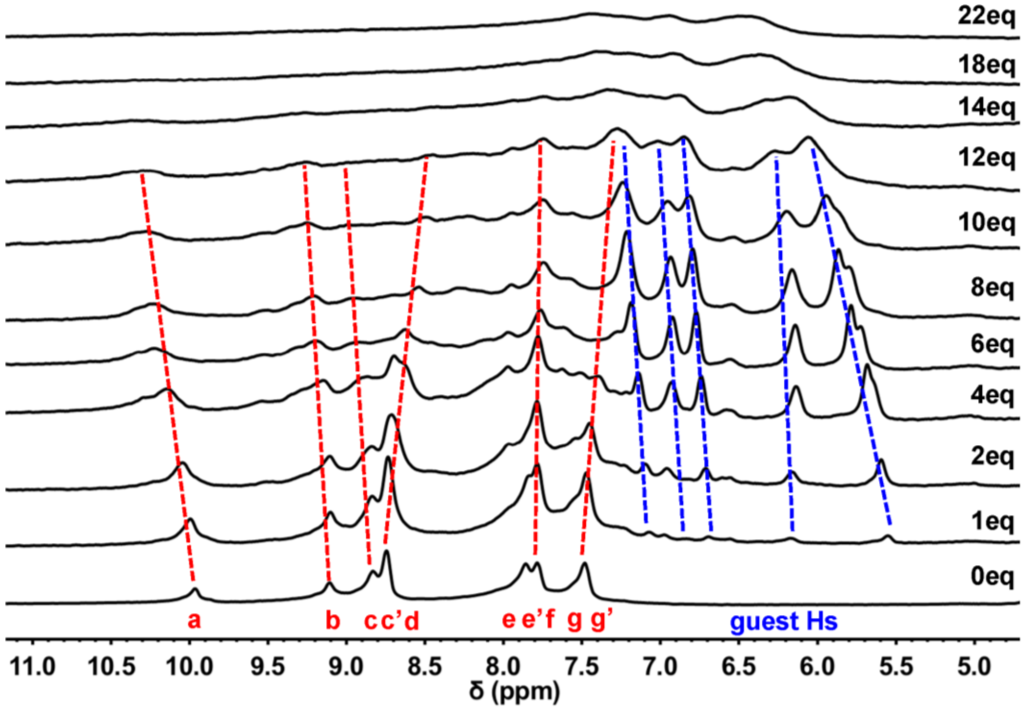

b

Supplementary Figure 27 | ^{1}H NMR titration. *S*-BINOL into ***Λ*-MOC-16** (DMSO-d_6/D_2O = 1/5) at 298 K **(a)** and 353 K **(b)**. Red lines show host protons while blue lines show guest protons.

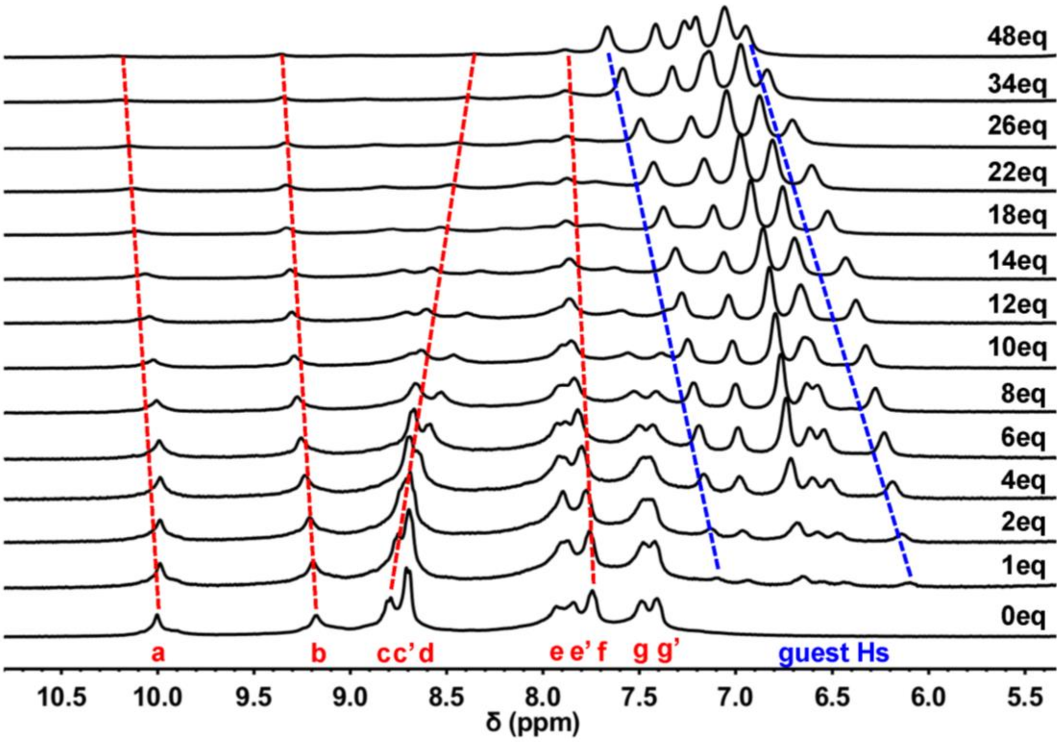

a

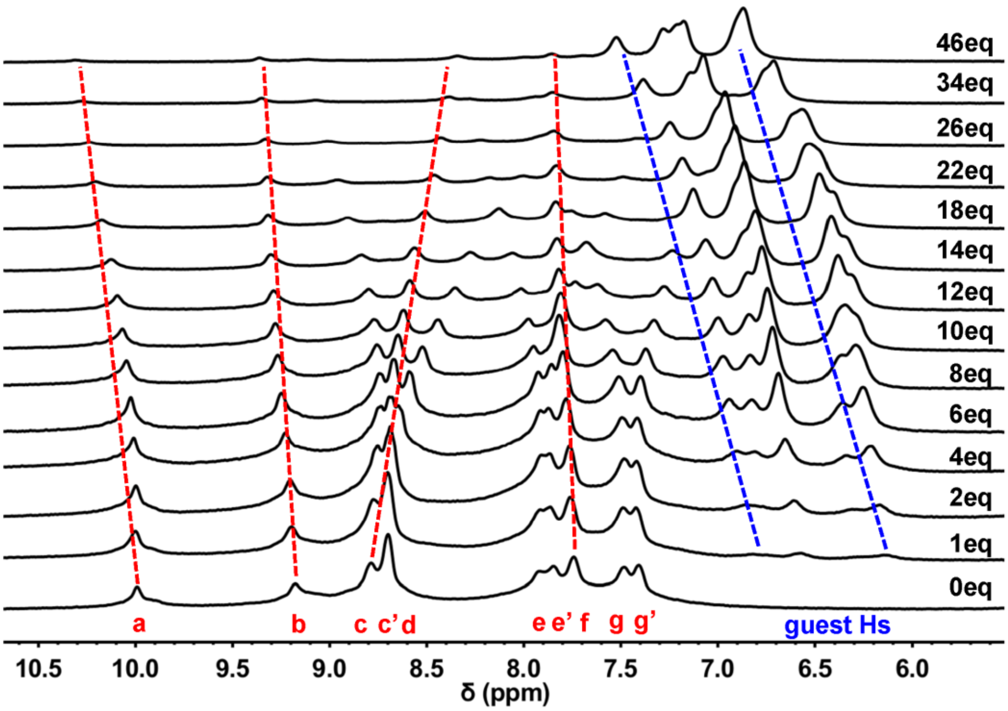

b

Supplementary Figure 28 | ^{1}H NMR titration in a mixture of DMSO-d_6/D_2O = 1/5 at 298 K. (a) *S*-1-(1-naphthyl)ethanol into ***Λ*-MOC-16**, **(b)** *S*-1-(1-naphthyl)ethanol into ***Δ*-MOC-16**. Red lines show host protons while blue lines show guest protons.

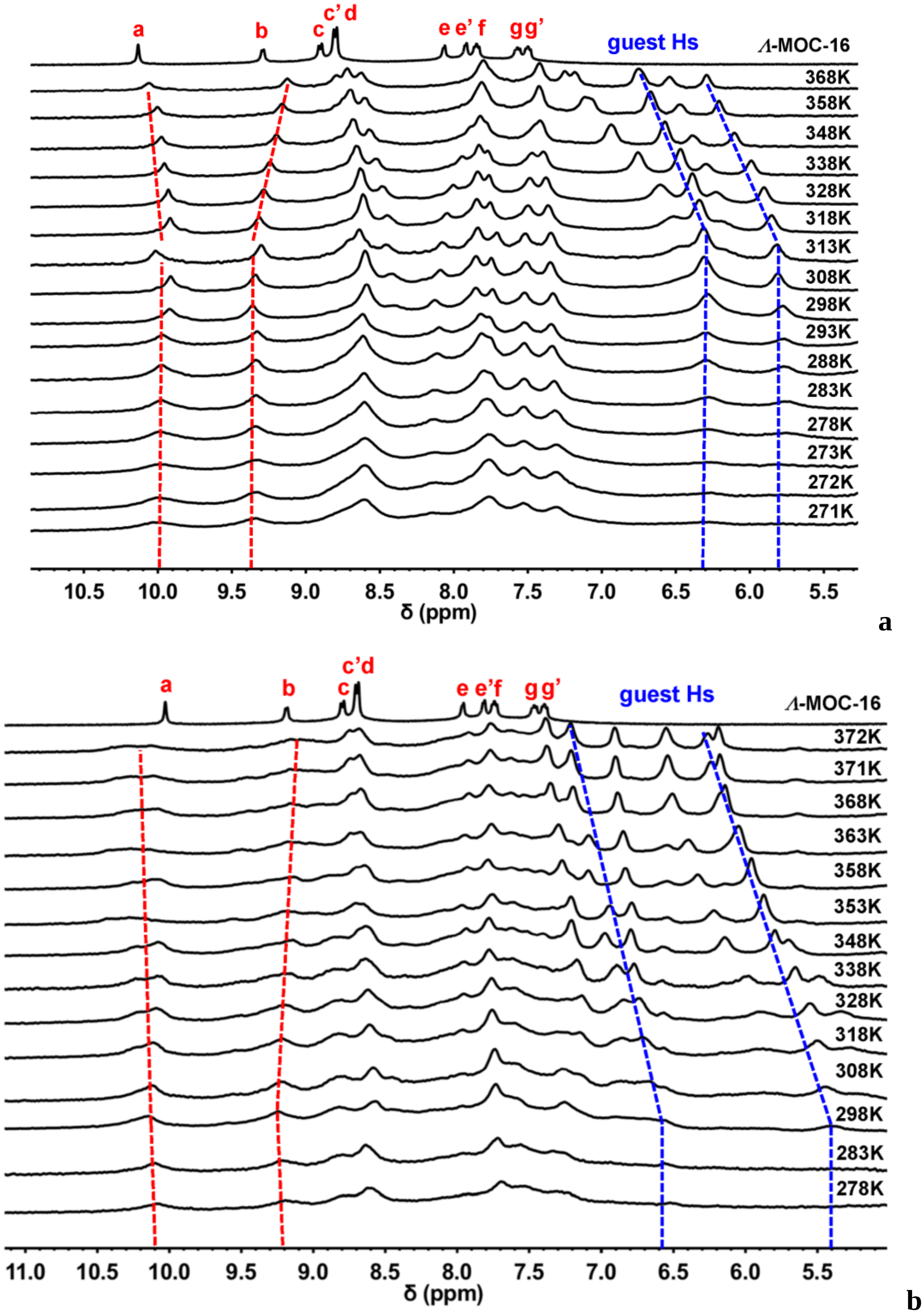

Supplementary Figure 29 | VT- ^{1}H NMR study of *R*-BINOL ⊂ *Λ*-MOC-16 (a) and ***S*-BINOL ⊂ *Λ*-MOC-16 (b)** in a mixture of DMSO-d_6/D_2O = 1/5. Red lines show host protons while blue lines show guest protons.

Supplementary Tables

Supplementary Table 1 | **Recycle test of chiral resolution results of ***R*/*S*-6-Br-BINOL racemate with ***Δ*-MOC-16**

	Solvent	Temp.	Time (h)	% ee within ***Δ*-MOC-16(*R*)**
fresh	D_2O/Et_2O	r.t.	1	57
cycle 1	D_2O/Et_2O	r.t.	1	55
cycle 2	D_2O/Et_2O	r.t.	1	50
cycle 3	D_2O/Et_2O	r.t.	1	52
cycle 4	D_2O/Et_2O	r.t.	1	51

Supplementary Table 2 │ Time-dependent chiral resolution results of ***R*/*S*-BINOL** racemate with ***Λ*-MOC-16.**

	Solvent	Temp.	Time (h)	% ee within ***Λ*-MOC-16(*S*)**
1	D_2O/Et_2O	r.t.	1	24
2	D_2O/Et_2O	r.t.	1.5	30
3	D_2O/Et_2O	r.t.	2	38
4	D_2O/Et_2O	r.t.	4	34
5	D_2O/Et_2O	r.t.	8	35
6	D_2O/Et_2O	r.t.	12	34

Supplementary Table 3 | Time-dependent chiral resolution results of *R*/*S*-BINOL racemate with *Δ*-MOC-16.

	Solvent	Temp.	Time (h)	% ee within **_Δ_-MOC-16(_R_)**
1	D_2O/Et_2O	r.t.	1	24
2	D_2O/Et_2O	r.t.	1.5	29
3	D_2O/Et_2O	r.t.	2	36
4	D_2O/Et_2O	r.t.	4	35
5	D_2O/Et_2O	r.t.	8	32
6	D_2O/Et_2O	r.t.	12	34

Supplementary Table 4 | Selected bond lengths (Å) and bond angles (°) for ***Δ*-1**.

Ru(1)-N(4)	2.058(6)	Ru(2)-N(12)	2.056(6)
Ru(1)-N(1)	2.063(6)	Ru(2)-N(10)	2.060(6)
Ru(1)-N(2)	2.065(6)	Ru(2)-N(11)	2.061(7)
Ru(1)-N(6)	2.066(6)	Ru(2)-N(9)	2.067(6)
Ru(1)-N(5)	2.066(7)	Ru(2)-N(7)	2.070(6)
Ru(1)-N(3)	2.069(6)	Ru(2)-N(8)	2.073(7)
N(4)-Ru(1)-N(1)	173.8(3)	N(12)-Ru(2)-N(10)	97.1(3)
N(4)-Ru(1)-N(2)	96.2(3)	N(12)-Ru(2)-N(11)	79.9(3)
N(1)-Ru(1)-N(2)	80.2(3)	N(10)-Ru(2)-N(11)	87.6(3)
N(4)-Ru(1)-N(6)	96.5(2)	N(12)-Ru(2)-N(9)	174.3(3)
N(1)-Ru(1)-N(6)	88.8(2)	N(10)-Ru(2)-N(9)	79.9(2)
N(2)-Ru(1)-N(6)	92.7(3)	N(11)-Ru(2)-N(9)	95.1(3)
N(4)-Ru(1)-N(5)	89.6(3)	N(12)-Ru(2)-N(7)	87.0(3)
N(1)-Ru(1)-N(5)	94.6(3)	N(10)-Ru(2)-N(7)	173.8(3)
N(2)-Ru(1)-N(5)	171.1(2)	N(11)-Ru(2)-N(7)	97.8(3)
N(6)-Ru(1)-N(5)	79.8(3)	N(9)-Ru(2)-N(7)	96.4(2)
N(4)-Ru(1)-N(3)	80.3(2)	N(12)-Ru(2)-N(8)	96.7(3)
N(1)-Ru(1)-N(3)	94.6(2)	N(10)-Ru(2)-N(8)	95.1(3)
N(2)-Ru(1)-N(3)	91.6(2)	N(11)-Ru(2)-N(8)	175.9(2)
N(6)-Ru(1)-N(3)	174.9(3)	N(9)-Ru(2)-N(8)	88.4(3)
N(5)-Ru(1)-N(3)	96.1(3)	N(7)-Ru(2)-N(8)	79.8(3)

Supplementary Table 5 | Selected bond lengths (Å) and bond angles (°) for ***Λ*-1**.

Ru(1)-N(4)	2.060(4)	Ru(2)-N(11)	2.054(4)
Ru(1)-N(2)	2.061(4)	Ru(2)-N(12)	2.054(4)
Ru(1)-N(1)	2.061(4)	Ru(2)-N(9)	2.059(4)
Ru(1)-N(5)	2.063(4)	Ru(2)-N(10)	2.061(4)
Ru(1)-N(6)	2.067(4)	Ru(2)-N(8)	2.067(4)
Ru(1)-N(3)	2.071(4)	Ru(2)-N(7)	2.069(4)
N(4)-Ru(1)-N(2)	95.93(17)	N(11)-Ru(2)-N(12)	80.09(18)
N(4)-Ru(1)-N(1)	173.74(16)	N(11)-Ru(2)-N(9)	94.97(16)
N(2)-Ru(1)-N(1)	80.12(18)	N(12)-Ru(2)-N(9)	174.37(18)
N(4)-Ru(1)-N(5)	89.81(17)	N(11)-Ru(2)-N(10)	87.59(16)
N(2)-Ru(1)-N(5)	171.24(15)	N(12)-Ru(2)-N(10)	97.10(16)
N(1)-Ru(1)-N(5)	94.69(17)	N(9)-Ru(2)-N(10)	79.89(15)
N(4)-Ru(1)-N(6)	96.24(15)	N(11)-Ru(2)-N(8)	175.98(15)
N(2)-Ru(1)-N(6)	92.74(16)	N(12)-Ru(2)-N(8)	96.57(18)
N(1)-Ru(1)-N(6)	88.83(15)	N(9)-Ru(2)-N(8)	88.47(16)
N(5)-Ru(1)-N(6)	80.03(17)	N(10)-Ru(2)-N(8)	95.09(16)
N(4)-Ru(1)-N(3)	80.38(15)	N(11)-Ru(2)-N(7)	97.84(16)
N(2)-Ru(1)-N(3)	91.50(16)	N(12)-Ru(2)-N(7)	86.99(16)
N(1)-Ru(1)-N(3)	94.80(15)	N(9)-Ru(2)-N(7)	96.41(15)
N(5)-Ru(1)-N(3)	95.99(16)	N(10)-Ru(2)-N(7)	173.71(17)
N(6)-Ru(1)-N(3)	174.84(17)	N(8)-Ru(2)-N(7)	79.66(17)

Supplementary Table 6 | Selected bond lengths (Å) and bond angles (˚) for ***Δ*-2**.

Ru(1)-N(5)	2.053(5)	Ru(1)-N(3)	2.070(5)
Ru(1)-N(2)	2.068(5)	Ru(1)-N(1)	2.071(5)
Ru(1)-N(6)	2.069(5)	Ru(1)-N(4)	2.072(5)
N(5)-Ru(1)-N(2)	95.3(2)	N(6)-Ru(1)-N(1)	100.5(2)
N(5)-Ru(1)-N(6)	79.3(2)	N(3)-Ru(1)-N(1)	85.2(2)
N(2)-Ru(1)-N(6)	88.8(2)	N(5)-Ru(1)-N(4)	87.46(19)
N(5)-Ru(1)-N(3)	95.6(2)	N(2)-Ru(1)-N(4)	175.9(2)
N(2)-Ru(1)-N(3)	97.5(2)	N(6)-Ru(1)-N(4)	94.7(2)
N(6)-Ru(1)-N(3)	172.22(19)	N(3)-Ru(1)-N(4)	79.1(2)
N(5)-Ru(1)-N(1)	173.9(2)	N(1)-Ru(1)-N(4)	98.6(2)
N(2)-Ru(1)-N(1)	78.55(19)		

Supplementary Table 7 | ** Selected bond lengths (Å) and bond angles (°) for ***Λ*-2.

Ru(1)-N(5)	2.061(5)	Ru(1)-N(3)	2.070(5)
Ru(1)-N(2)	2.063(5)	Ru(1)-N(4)	2.077(5)
Ru(1)-N(6)	2.067(4)	Ru(1)-N(1)	2.078(5)
N(5)-Ru(1)-N(2)	95.19(19)	N(6)-Ru(1)-N(4)	94.82(18)
N(5)-Ru(1)-N(6)	79.39(18)	N(3)-Ru(1)-N(4)	79.08(19)
N(2)-Ru(1)-N(6)	88.80(18)	N(5)-Ru(1)-N(1)	173.86(19)
N(5)-Ru(1)-N(3)	95.48(19)	N(2)-Ru(1)-N(1)	78.68(18)
N(2)-Ru(1)-N(3)	97.49(19)	N(6)-Ru(1)-N(1)	100.54(18)
N(6)-Ru(1)-N(3)	172.26(18)	N(3)-Ru(1)-N(1)	85.18(18)
N(5)-Ru(1)-N(4)	87.60(17)	N(4)-Ru(1)-N(1)	98.51(19)
N(2)-Ru(1)-N(4)	175.79(19)		

Supplementary Table 8 | **Selected bond lengths (Å) and bond angles (°) for ***Δ*-MOC-16.

Pd(1)-N(5)#1	2.019(10)	Pd(2)-N(15)#1	2.037(9)
Pd(1)-N(5)#2	2.019(10)	Ru(1)-N(6)	2.005(7)
Pd(1)-N(5)#3	2.019(10)	Ru(1)-N(2)	2.019(8)
Pd(1)-N(5)	2.019(10)	Ru(1)-N(11)	2.050(6)
Pd(2)-N(10)	1.999(8)	Ru(1)-N(7)	2.052(4)
Pd(2)-N(10)#4	1.999(8)	Ru(1)-N(1)	2.056(4)
Pd(2)-N(15)#5	2.037(9)	Ru(1)-N(12)	2.063(5)
N(5)#1-Pd(1)-N(5)#2	177.2(4)	N(2)-Ru(1)-N(11)	174.0(2)
N(5)#1-Pd(1)-N(5)#3	89.965(11)	N(6)-Ru(1)-N(7)	78.5(2)
N(5)#2-Pd(1)-N(5)#3	89.965(11)	N(2)-Ru(1)-N(7)	94.0(2)
N(5)#1-Pd(1)-N(5)	89.965(11)	N(11)-Ru(1)-N(7)	90.5(2)
N(5)#2-Pd(1)-N(5)	89.965(11)	N(6)-Ru(1)-N(1)	95.4(2)
N(5)#3-Pd(1)-N(5)	177.2(4)	N(2)-Ru(1)-N(1)	80.0(2)
N(10)-Pd(2)-N(10)#4	178.2(4)	N(11)-Ru(1)-N(1)	96.0(2)
N(10)-Pd(2)-N(15)#5	91.1(3)	N(7)-Ru(1)-N(1)	171.5(2)
N(10)#4-Pd(2)-N(15)#5	88.9(3)	N(6)-Ru(1)-N(12)	172.9(2)
N(10)-Pd(2)-N(15)#1	88.9(3)	N(2)-Ru(1)-N(12)	95.6(2)
N(10)#4-Pd(2)-N(15)#1	91.1(3)	N(11)-Ru(1)-N(12)	79.9(2)
N(15)#5-Pd(2)-N(15)#1	179.7(5)	N(7)-Ru(1)-N(12)	96.62(18)
N(6)-Ru(1)-N(2)	89.8(3)	N(1)-Ru(1)-N(12)	89.97(19)
N(6)-Ru(1)-N(11)	95.0(3)		

Symmetry transformations used to generate equivalent atoms:

#1 y,-x+1,z #2 -y+1,x,z #3 -x+1,-y+1,z

#4 y,x,-z+2 #5 -x+1,y,-z+2 #6 y-1/2,x+1/2,-z+3/2

Supplementary Table 9 | **Selected bond lengths (Å) and bond angles (°) for ***Λ*-MOC-16.

Pd(1)-N(5)	1.966(9)	Pd(2)-N(15)#5	2.008(9)
Pd(1)-N(5)#1	1.967(9)	Ru(1)-N(2)	2.019(10)
Pd(1)-N(5)#2	1.967(10)	Ru(1)-N(11)	2.032(7)
Pd(1)-N(5)#3	1.967(9)	Ru(1)-N(12)	2.045(6)
Pd(2)-N(10)#4	2.002(8)	Ru(1)-N(1)	2.058(6)
Pd(2)-N(10)	2.002(8)	Ru(1)-N(6)	2.071(9)
Pd(2)-N(15)#1	2.008(9)	Ru(1)-N(7)	2.087(6)
N(5)-Pd(1)-N(5)#1	89.997(7)	N(11)-Ru(1)-N(12)	79.0(3)
N(5)-Pd(1)-N(5)#2	89.993(7)	N(2)-Ru(1)-N(1)	79.2(3)
N(5)#1-Pd(1)-N(5)#2	178.9(5)	N(11)-Ru(1)-N(1)	96.3(2)
N(5)-Pd(1)-N(5)#3	178.9(5)	N(12)-Ru(1)-N(1)	90.2(2)
N(5)#1-Pd(1)-N(5)#3	89.995(6)	N(2)-Ru(1)-N(6)	90.2(4)
N(5)#2-Pd(1)-N(5)#3	89.995(6)	N(11)-Ru(1)-N(6)	94.6(3)
N(10)#4-Pd(2)-N(10)	180.0(6)	N(12)-Ru(1)-N(6)	171.8(3)
N(10)#4-Pd(2)-N(15)#1	91.8(3)	N(1)-Ru(1)-N(6)	95.7(3)
N(10)-Pd(2)-N(15)#1	88.1(3)	N(2)-Ru(1)-N(7)	93.7(3)
N(10)#4-Pd(2)-N(15)#5	88.2(3)	N(11)-Ru(1)-N(7)	91.1(3)
N(10)-Pd(2)-N(15)#5	91.9(3)	N(12)-Ru(1)-N(7)	95.6(2)
N(15)#1-Pd(2)-N(15)#5	178.5(5)	N(1)-Ru(1)-N(7)	171.4(2)
N(2)-Ru(1)-N(11)	173.7(3)	N(6)-Ru(1)-N(7)	79.2(3)
N(2)-Ru(1)-N(12)	96.5(3)		

Symmetry transformations used to generate equivalent atoms:

#1 y,-x+1,z #2 -y+1,x,z #3 -x+1,-y+1,z

#4 y,x,-z #5 -x+1,y,-z #6 y+1/2,x-1/2,-z+1/2

Supplementary Methods

1. General methods

Unless otherwise stated, all commercial reagents and solvents were used as commercially purchased without additional purification. The ^{1}H NMR、COSY spectra were recorded on Bruker AVANCE III 400 (400 MHz). Circular dichroism spectra and UV-vis absorption spectra were measured with a JASCO J-810 spectropolarimeter. Specific rotations were measured on ADP440+B+S. HR-ESI-TOF mass spectra were tested on Bruker Maxis 4G and data analyses were processed on Bruker Data Analysis software. HPLC spectra were measured on Agilent-2000.

2. X-Ray single-crystal and powder diffraction

(1) *Δ*-1 and *Λ*-1

Diffraction data for two single crystals of ***Δ*-** and ***Λ*-1-PF$_6$** were collected on an Agilent SuperNova X-Ray diffractometer using micro-focus X-ray sources (Mo Kα, λ = 0.71073 Å). The structures were solved by direct methods and refined by full-matrix least squares against F^2 of all data using the SHELXTL-2014 program package.

***Δ*-1-PF$_6$** crystallizes in the chiral space groups *P*4(1) with a chemical composition of $[Ru(Phen)_3]_2(PF_6)_4(C_6H_5CH_3)(CH_3CN)_2$in the asymmetric unit. Two $[Ru(Phen)_3]^{2+}$ motifs have the same absolute *Δ*-configuration. The Flack parameter was refined as 0.00(4) by classical fit to all intensities, conforming the correct chirality in agreement with the experimental synthesis. The solvated toluene molecules are disordered over two positions and treated isotropically with the free-variable occupancy. The benzene ring was modelled by AFIX 66 and the methyl group was fixed by SADI. FLAT was used to constrain all toluene atoms as planar. SAME is used to simulate the PART 2 as those in PART 1. Totally 21 restraints were introduced.

***Λ*-1-PF$_6$** crystallizes in the chiral space groups *P*4(3) with the same chemical composition of $[Ru(Phen)_3]_2(PF_6)_4(C_6H_5CH_3)(CH_3CN)_2$ as ***Δ*-1-PF$_6$** in the asymmetric unit, but showing the opposite *Λ*-configuration for two $[Ru(Phen)_3]^{2+}$ motifs. The Flack parameter was determined as -0.033(8) using 7351 quotients [(I+)-(I-)]/[(I+)+(I-)] (Parsons, Flack and

Wagner, Acta Cryst. B69 (2013) 249-259), which is close to zero thereof indicates the correct chirality based on the synthetic materials. The solvated toluene molecules are disordered over two positions and treated by modelling the benzene rings with AFIX 66. The methyl groups were fixed by DFIX. FLAT was used to constrain all toluene atoms being planar. ISOR was applied to all the disordered toluene atoms. Totally 99 restraints were resulted.

(2) *Δ*-2 and *Λ*-2

Diffraction data for two single crystals of ***Δ*-2-ClO$_4$** and ***Λ*-2-ClO$_4$** were collected on an Agilent SuperNova X-Ray diffractometer using micro-focus X-ray sources (Cu Kα, λ = 1.54178 Å). The structures were solved by direct methods and refined by full-matrix least squares against F^2 of all data using the SHELXTL-2014 program package.

Both ***Δ*-2-ClO$_4$** and ***Λ*-2-ClO$_4$** crystallize in the chiral space groups *P*2(1)2(1)2(1) with the same chemical composition of [Ru(Phendione)$_3$](ClO$_4$)$_2$(H$_2$O)(CH$_3$CN)$_2$ in the asymmetric unit. However, ***Δ*-2-ClO$_4$** shows the absolute *Δ*-configuration of [Ru(Phendione)$_3$]$^{2+}$ cation while that of ***Λ*-2-ClO$_4$** shows the opposite *Λ*-configuration. The Flack parameter for ***Δ*-2-ClO$_4$** was determined s -0.015(4) using 3147 quotients [(I+)-(I-)]/[(I+)+(I-)] (Parsons, Flack and Wagner, Acta Cryst. B69 (2013) 249-259), while that for ***Λ*-2-ClO$_4$** was determined as -0.011(3) using 3295 quotients. Both Flack values are close to zero, indicating the correct chirality refined for the single crystals, and in agreement with the experimental syntheses.

For ***Δ*-2-ClO$_4$**, the tiny needle crystal gives relative weak diffractions although Cu radiation was applied. Therefore, all solvated MeCN and water molecules, swell as oxygen atoms of perchlorate anions were refined isotropically to give moderate data/parameter ratio (8.72). The water molecule is disordered over two positions and treated as fractional occupancy with free variate. H atoms of water molecules were not calculated. Two long SP^2 C-C bond distances were restrained by DFIX to give realistic bond lengths. O3 atom shows a little unusual ellipsoid shape, thereof was refined with ISOR. Total 8 restraints were introduced. In the crystal lattice, there are some intermolecular O..C and N...C contacts, which are owing to pi...pi, anion...pi and MeCN...pi interactions. Two level B alerts of short interactions between O2w...O3 and O2w...O9 appear because the H atoms of O2w were not added. For ***Λ*-2-ClO$_4$**, similar crystallographic problems were encountered due to poor reflection intensities

(data/parameter ratio of 8.72). Therefore, similar refinement constraints were applied as in ***Δ*-2-ClO$_4$**. H atoms of the solvated MeCN were also neglected.

(3) *Δ*-MOC-16 and *Λ*-MOC-16

Single crystals suitable for X-ray diffraction were obtained by co-crystallization of ***Δ*-MOC-16** with ***S*-BINOL** or ***Λ*-MOC-16** with ***R*-BINOL**, respectively, in MeCN solution by diffusion of isopropyl ether for 2 weeks. Diffraction data were collected on an Agilent SuperNova X-Ray diffractometer using micro-focus X-ray sources (Cu Kα, λ = 1.54184 Å). The structures were solved by direct methods and refined by full-matrix least squares against F^2 of all data using the SHELXTL-2014 program package.

Both ***Δ*-MOC-16** and ***Λ*-MOC-16** crystallize in the chiral space groups *I*422. The asymmetric unit contains one eighth of **$[Pd_6(RuL_3)_8]^{28+}$** cationic cage, a half of BINOL guest molecule, counter anions and solvent molecules. Due to the huge hollow cage molecules which pack in the crystal lattice to result in large voids inside and outside the cages where the solvent molecules and counter anions are disorderedly distributed, only week diffraction data (especially in high theta angles) were obtained although Cu radiation was applied. Therefore, the disordered solvents and anions could not be unambiguously determined and were removed in the final refinement, and the contribution of their scattering to the intensity of the reflections is calculated and accounted for using SQUEEZE. Nevertheless, the Flack parameter for ***Δ*-MOC-16** was determined as 0.131(13) using BASF/TWIN with an inversion twin refinement, while that of ***Λ*-MOC-16** was determined as 0.139(16). Both Flack values are close to zero, indicating the correct chirality refined for the single crystals, and in agreement with the chiral metalloligands and BINOLs used in the experimental syntheses.

For ***Δ*-MOC-16**, the reflections with the resolution below 1.0 Å give high *R*(int) value (> 0.45). The overall poor diffractions cause anisotropic refinement of non-H atoms with relatively nonideal displacement parameters Ueq, of which all atoms on BINOL guest molecules are treated by SIMU/DILU, in together with other 22 C-atom and 5 N-atoms on the cage. DIFX is used for a few unreasonable C-C and C-O bonds, and AFIX 66 is used for three aromatic rings. SAME is used to simulate part of fragment of one ligand with the other. These refinement constraints cause totally 221 restraints and some C-level alerts of large hirshfeld

difference which was not further improved. The B-level alert of D-H without acceptor for O1-H1A is due to removal of possible donor atoms from solvents or anions.

For **Λ-MOC-16**, the reflections are even weak with the resolution below 1.2 Å giving high *R*(int) value (> 0.45) and a low ratio of observed/unique reflections (45 %) obtained. The similar crystallographic problems as in **Δ-MOC-16** were treated in similar ways. These refinement constraints cause totally 252 restraints and some C-level alerts of large hirshfeld which was not further improved. The B-level alert of D-H without acceptor for O1-H1A is due to removal of possible donor atoms from solvents or anions.

3. Syntheses of enantiopure metalloligands and Δ- and Λ-MOC-16

3.1 Synthesis of *rac*-1 [(Ru(Phen)$_3$]Cl_2·xH_2O)

$Ru(DMSO)_4Cl_2$ (258 mg, 0.53 mmol), 1,10-phenanthroline (**Phen**, 364 mg, 1.84 mmol) were dissolved in 30 mL of ethanol. The solution was stirred and refluxed for 20 h during which the color turned from yellow to deep red. After cooling the reaction, the solvent was removed by rotary evaporator. The crude product was washed with 10 mL of benzene to get rid of the residual Phen. The deep red solid product was obtained after filtration and dried under vacuum. Yield: 350 mg (92 %). ^1H NMR (400 MHz, DMSO-d_6): δ 8.78 (dd, J = 8.2, 1.1 Hz, 6H), 8.39 (s, 6H), 8.08 (dd, J = 5.2, 1.1 Hz, 6H), 7.77 (dd, J = 8.2, 5.3 Hz, 6H).
ESI$^+$-MS: *m/z* calcd. for $C_{36}H_{24}N_6Ru$ $[Ru(Phen)_3]^{2+}$ 321.0552, found 321.0944.

3.2 Synthesis of $K_2[Sb_2\{(+)\text{-tartrate}\}_2]\cdot 3H_2O$

In a 100 mL round-bottom flask, L-(+)-tartaric acid (0.76 g, 5.1 mmol), KOH (0.28 g, 5.0 mmol), and deionized water (50 mL) were added. After being stirred for 20 min at 70 ^{o}C, the supernatant solution was obtained. To this solution was added Sb_2O_3 (0.73 g, 5.0 mmol) powder and the reaction mixture was stirred at 100 ^{o}C for 3 days. After filtration, the water was rotary evaporated to give the concentrate which was standing for three days to afford crystal product. Yield: 1.20 g (71 %).

3.3 Resolution of Δ-1 and Λ-1

(1) Δ-[Ru(Phen)$_3$](PF$_6$)$_2$ (Δ-1-PF$_6$). Compound ***rac*-1** (356 mg, 0.500 mmol) was dissolved in 25 mL water at 70 ^{o}C. To this solution was added 13 mL of hot water solution of $K_2[Sb_2\{(+)\text{-tartrate}\}_2]\cdot 3H_2O$ (334 mg, 0.500 mmol). Then, the light yellow precipitate

appeared quickly. After adding 10 ml water additionally, the solution was allowed to stir at 70 ^{o}C for 1 h, then cooled to room temperature and stayed overnight. The orange-yellow precipitation was filtered as *Λ*-[Ru(Phen)$_3$] {Sb$_2${(+)-tartrate}$_2$] (283 mg) for use in the next step. To the collected filtrate was added 1 mL of saturated KPF$_6$ aqueous. The red precipitation was afforded, then filtered and dried under vacuum. The resulting product was obtained as ***Δ*-[Ru(Phen)$_3$](PF$_6$)$_2$ (*Δ*-1-PF$_6$).** Yield: 205 mg (90 %). ^{1}H NMR (400 MHz, DMSO-*d$_6$*): *δ* 8.77 (dd, *J* = 8.3, 1.1 Hz, 6H), 8.38 (s, 6H), 8.08 (dd, *J* = 5.2, 1.1 Hz, 6H), 7.76 (dd, *J* = 8.2, 5.3 Hz, 6H). ESI$^+$-MS: *m/z* calcd for $C_{36}H_{24}N_6Ru$ [Ru(Phen)$_3$]$^{2+}$ 321.0552, found 321.0534; *m/z* calcd for $C_{36}H_{24}N_6PF_6Ru$ [Ru(Phen)$_3$(PF$_6$)]$^+$ 787.0752, found 787.0717. [α]^{30}D = -1138 °, c = 1.0, MeCN.

(2) *Λ*-[Ru(Phen)$_3$](PF$_6$)$_2$ (*Λ*-1-PF$_6$). *Λ*-[Ru(Phen)$_3$] {Sb$_2${(+)-tartrate}$_2$] (283 mg) was added in 100 mL of 0.05 M NaOH aqueous. After being stirred for 5 min, a white solid was precipitated and filtered. To the collected filtrate was added 1 mL of saturated KPF$_6$ aqueous. The orange-red solid was precipitated out of the solution. After filtration and dry under vacuum, the resulting product was obtained as ***Λ*-[Ru(Phen)$_3$](PF$_6$)$_2$ (*Λ*-1-PF$_6$).** Yield: 200 mg (88 %). ^{1}H NMR (400 MHz, DMSO-*d$_6$*): *δ* 8.77 (dd, *J* = 8.3, 1.2 Hz, 6H), 8.38 (s, 6H), 8.08 (dd, *J* = 5.2, 1.1 Hz, 6H), 7.76 (dd, *J* = 8.2, 5.3 Hz, 6H). ESI$^+$-MS: *m/z* calcd for $C_{36}H_{24}N_6Ru$ [Ru(Phen)$_3$]$^{2+}$ 321.0552, found 321.0547; *m/z* calcd for $C_{36}H_{24}N_6PF_6Ru$ [Ru(Phen)$_3$(PF$_6$)]$^+$ 787.0752, found 787.0724. [α]^{30}D = 1136 °, c = 1.0, MeCN.

We used two kinds of methods to prove the chiral purity of ***Δ*-** and ***Λ*-1-PF$_6$**, i.e., single crystal structural determination and detection of ^{1}H NMR signal response applying chiral shift reagents.

<u>Method I</u>: Orange needle like single crystals of ***Δ*-** and ***Λ*-1-PF$_6$** were grown from natural evaporation of acetonitrile/toluene (v:v = 1:1) solution, which crystallize in a pair of chiral space groups *P*4(1) and *P*4(3), respectively. ***Δ*-1-PF$_6$** crystal shows a chemical composition of [Ru(Phen)$_3$]$_2$(PF$_6$)$_4$($C_6H_5CH_3$)(CH_3CN)$_2$ in the asymmetric unit, in which two [Ru(Phen)$_3$]$^{2+}$ motifs have the same absolute *Δ*-configuration. The Flack parameter of 0.00(4) was obtained in the final refinement, conforming the correct chirality in agreement with the experimental synthesis. ***Λ*-1-PF$_6$** crystal is essentially the same as ***Δ*-1-PF$_6$** only with the opposite handedness. The Flack parameter of -0.033(8) was obtained from 7349 selected quotients

(Parsons' method), conforming the correct chirality in agreement with the experimental synthesis. For both enantiomers, the phase purity of the bulk sample has been verified by the powder X-ray diffraction measurements.

Method II: In order to test the chiral purity, the anions in ***Δ/Λ*-1-PF$_6$** were convereted into Cl^- for better chiral shift signals [A. S. Torres, D. J. Maloney, D. Tate, Y. Saad, F. M. MacDonnell, *Inorg. Chim. Acta*. **1999**, 293, 37.]:

(1) *Δ*-[Ru(phen)$_3$]Cl$_2$ (*Δ*-1-Cl). *Δ*-[Ru(phen)$_3$](PF$_6$)$_2$ (*Δ*-1-PF$_6$, 100 mg) was converted to the chloride salts by precipitation upon addition of an acetone solution (2 mL) of ^{n}Bu$_4$NCl (100 mg). The precipitate was centrifuged, washed with acetone and diethyl ether twice, dried. Yield 80%. Optical purity 94.8% as determined from integration analysis using chiral shift reagent Eu((+)tfc)$_3$. ^{1}H NMR (400 MHz, CD$_2$Cl$_2$): δ 8.62 (d, *J* = 8.1 Hz, 6H), 8.29 (d, *J* = 4.7 Hz, 6H), 8.25 (s, 6H), 7.86 (dd, *J* = 7.9, 5.2 Hz, 6H).

(2) *Λ*-[Ru(phen)$_3$]Cl$_2$ (*Λ*-1-Cl). Compound ***Λ*-[Ru(phen)$_3$]Cl$_2$** was prepared by the procedure detailed above for ***Δ*-[Ru(phen)$_3$]Cl$_2$. *Λ*-[Ru(phen)$_3$](PF$_6$)$_2$ (*Λ*-1-PF$_6$**) was used as starting material instead of compound ***Δ*-[Ru(phen)$_3$](PF$_6$)$_2$**. Yield 84%. Optical purity 95.3% as determined from integration analysis. ^{1}H NMR (400 MHz, CD$_2$Cl$_2$): δ 8.62 (d, *J* = 8.1 Hz, 6H), 8.29 (d, *J* = 4.8 Hz, 6H), 8.25 (s, 6H), 7.86 (dd, *J* = 8.1, 5.2 Hz, 6H).

3.4 Synthesis of *Δ*-2 and *Λ*-2 (*Δ*-/*Λ*-[Ru(Phendione)$_3$]$^{2+}$)

(1) *Δ*-Ru[(Phendione)$_3$](PF$_6$)$_2$ (*Δ*-2-PF$_6$, phendione = 1,10-Phenanthroline-5,6-Dione**).** In a 20 mL of round-bottom flask, *Δ*-Ru(Phen)$_3$(PF$_6$)$_2$ (100 mg, 0.1 mmol), KBr (100 mg, 0.8 mmol), 1 mL of 65% HNO$_3$ and 2 mL of 98% H$_2$SO$_4$ were added stepwisely. The reaction mixture was stirred at 100 ^{o}C for 5 h to afford a dark-green solution. 3 g NaOH in 3 mL of water was dropped carefully to the solution to adjust pH = 4 at 0 ^{o}C. A dark brown precipitate formed immediately. After centrifugation, the crude product was collected, extracted with 10 mL of methanol twice and the extracts were combined. The solvent was removed under reduced pressure to afford the desired product. Yield: 90 mg (82 %). ^{1}H NMR (400 MHz, DMSO-*d$_6$*): δ 8.58 (dd, *J* = 7.9 Hz, 0.8 Hz, 6H), 8.02 (dd, *J* = 5.7 Hz, 1.0 Hz, 6H), 7.79 (dd, *J* = 8.2 Hz, 5.6 Hz, 6H). ESI$^+$-MS: *m/z* calcd for C$_{36}$H$_{18}$N$_6$O$_6$Ru [*Δ*-Ru(Phendione)$_3$]$^{2+}$ 366.5177, found 366.5180.

(2) Λ-[Ru(Phendione)$_3$](PF$_6$)$_2$ (Λ-2-PF$_6$). Compound Λ-[Ru(Phendione)$_3$]$^{2+}$ was prepared by a procedure similar to that described above for Δ-[Ru(Phendione)$_3$]$^{2+}$. Λ-[Ru(Phen)$_3$](PF$_6$)$_2$ (100 mg, 0.1mmol) was used instead of Δ-[Ru(Phen)$_3$](PF$_6$)$_2$. Yield: 95 mg (86%). ^{1}H NMR (400 MHz, DMSO-*d$_6$*): δ 8.58 (dd, *J* = 7.9 Hz, 1.2 Hz, 6H), 8.02 (dd, *J* = 5.6 Hz, 1.3 Hz, 6H), 7.79 (dd, *J* = 8.4 Hz, 5.6 Hz, 6H). ESI$^+$-MS: *m/z* calcd for $C_{36}H_{18}N_6O_6Ru$ [Λ-Ru(Phendione)$_3$]$^{2+}$ 366.5177, found 366.5168.

In order to obtain single crystals suitable for X-ray diffraction, Δ-[Ru(Phendione)$_3$](ClO$_4$)$_2$ and Λ-[Ru(Phendione)$_3$](ClO$_4$)$_2$ (**Δ-2-ClO$_4$** and **Λ-2-ClO$_4$**) were synthesized.

(3) Δ-[Ru(Phendione)$_3$](ClO$_4$)$_2$ (Δ-2-ClO$_4$). 5 mL cold 98% H_2SO_4 was added dropwise to **Δ-[Ru(Phen)$_3$](PF$_6$)$_2$** (0.5 g, 0.5 mmol) at 0 ^{o}C in a 25 mL flask and allowed to stir until **Δ-[Ru(Phen)$_3$](PF$_6$)$_2$** dissolved, followed by addition of 0.25 g NaBr. After warming to r.t., 2.5 mL 65% HNO_3 was added dropwise. The reaction mixture was stirred at 100 ^{o}C for 20 min to afford an olive-green solution. The heat was removed and the bromine vapors were removed by a stream of nitrogen. The solution was poured into a soluton of $NaClO_4$ (10 g) in 15 mL water and left to stand for 1 h. After dilution to 100 mL with water, the solution was left to stand overnight in a refrigerator. The precipitate was filtered and washed with water, then dried under vacuum to give a dark-black powder. Yield: 340 mg (73%). ^{1}H NMR (400 MHz, DMSO-*d$_6$*): δ 8.61 (d, *J* = 7.0 Hz, 6H), 7.97 (d, *J* = 4.7 Hz, 6H), 7.82 (dd, *J* = 7.9, 5.7 Hz, 6H). ESI$^+$-MS: *m/z* calcd for $C_{36}H_{18}N_6O_6Ru$ [Δ-Ru(Phendione)$_3$]$^{2+}$ 366.0165, found 366.0177; *m/z* calcd for $C_{36}H_{18}N_6O_6RuClO_4$ [Δ-Ru(Phendione)$_3$+ClO$_4^-$]$^+$ 830.9817, found 830.9832.

(4) Λ-[Ru(Phendione)$_3$](ClO$_4$)$_2$ (Λ-2-ClO$_4$). Compound **Λ-[Ru(Phendione)$_3$](ClO$_4$)$_2$** was prepared by the procedure detailed above for **Δ-[Ru(Phendione)$_3$](ClO$_4$)$_2$**. The enantiomer **Λ-[Ru(Phen)$_3$](PF$_6$)$_2$** was used as starting material instead of compound **Δ-[Ru(Phen)$_3$](PF$_6$)$_2$**. Yield: 337 mg (72 %). ^{1}H NMR (400 MHz, DMSO-*d$_6$*): δ 8.60 (dt, *J* = 6.2, 3.1 Hz, 6H), 7.97 (dd, *J* = 5.6, 1.0 Hz, 6H), 7.82 (dd, *J* = 7.9, 5.7 Hz, 6H). ESI$^+$-MS: *m/z* calcd for $C_{36}H_{18}N_6O_6Ru$ [Λ-Ru(Phendione)$_3$]$^{2+}$ 366.0165, found 366.0174; *m/z* calcd for $C_{36}H_{18}N_6O_6RuClO_4$ [Λ-Ru(Phendione)$_3$+ClO$_4^-$]$^+$ 830.9817, found 830.9839.

Dark black needle like single crystals of **Δ-2-ClO$_4$** and **Λ-2-ClO$_4$** were grown from the natural evaporation of saturated solution of MeCN, which crystallize in the chiral space groups *P*2(1)2(1)2(1). The the asymmetric unit of **Δ-2-ClO$_4$** crystal has a chemical

composition of [Ru(Phendione)$_3$](ClO$_4$)$_2$(H$_2$O)(CH$_3$CN)$_2$, in which the [Ru(Phendione)$_3$]$^{2+}$ cation shows the absolute ***Δ***-configuration. The Flack parameter of -0.018(4) was obtained from 3146 selected quotients (Parsons' method), conforming the correct chirality in agreement with the experimental synthesis. ***Λ*-2-ClO$_4$** crystal is essentially the same as ***Δ*-2-ClO$_4$** only with the opposite handedness. The Flack parameter of -0.011(4) was obtained from 3296 selected quotients (Parsons' method), also conforming the correct chirality in agreement with the experimental synthesis. For both enantiomers, the phase purity of the bulk sample has been verified by the powder X-ray diffraction measurements

3.5 Synthesis of *Δ*-3-PF$_6$ and *Λ*-3-PF$_6$ (*Δ*-/*Λ*-[Ru(L)$_3$](PF$_6$)$_2$)

(1) *Δ*-[Ru(L)$_3$](PF$_6$)$_2$ (*Δ*-3-PF$_6$, L= 2-(pyridin-3-yl)-1H-imidazo[4,5-f][1,10] phenanthroline**).** Compound ***Δ*-2-PF$_6$** (100 mg, 0.1 mmol), ammonium acetate (400 mg, 5.2 mmol) and 3-pyridinecarboxaldehyde (50 mg, 0.5 mmol) were dissolved in 4 mL of acetic acid. After being stirred at 100 ^{o}C for 6 h, the solution was poured into 4 mL of water and neutralized with ammonia to give brown precipitation. After centrifugation, the dark brown solid was washed with 5 mL of water twice and extracted with 10 mL of methanol. The solvent was removed by rotary evaporator. The resulting solid was dried under vacuum to afford the desired product. Yield: 15 mg (13%). ^{1}H NMR (400 MHz, DMSO-*d$_6$*): δ 9.53 (s, 3H), 8.97 (d, J = 8.0 Hz, 6H), 8.64 (d, J = 7.9 Hz, 3H), 8.58 (br, 3H), 7.88 (m, 6H), 7.69 (br, 6H), 7.53 (br, 3H). ESI$^+$-MS: *m/z* calcd for $C_{54}H_{33}N_{15}Ru$ {[Ru(L)$_3$]$^{2+}$} 496.6045, found 496.6032; *m/z* calcd for $C_{54}H_{32}N_{15}Ru$ {[*Δ*-Ru(L)$_3$-H$^+$]$^+$} 992.2017, found 992.1977; *m/z* calcd for $C_{54}H_{33}N_{15}PF_6Ru$ {[*Δ*-Ru(L)$_3$+PF$_6$]$^+$} 1138.1737, found 1138.1669.

(2) *Λ*-[Ru(L)$_3$](PF$_6$)$_2$ (*Λ*-3-PF$_6$). Compound ***Λ*-3-PF$_6$** was prepared by the procedure detailed above for ***Δ*-3-PF$_6$**. Compound ***Λ*-2-PF$_6$** was used as starting material instead of compound ***Δ*-2-PF$_6$**. Yield: 18 mg (15 %). ^{1}H NMR (400 MHz, DMSO-*d$_6$*): δ 9.52 (s, 3H), 8.96 (d, J = 7.8 Hz, 6H), 8.64 (d, J = 7.2 Hz, 3H), 8.57 (br, 3H), 7.88 (br, 6H), 7.68 (br, 6H), 7.52 (br, 3H). ESI$^+$-MS: *m/z* calcd for $C_{54}H_{33}N_{15}Ru$ {[*Λ*-Ru(L)$_3$]$^{2+}$} 496.6045, found 496.6040; *m/z* calcd for $C_{54}H_{32}N_{15}Ru$ {[*Λ*-Ru(L)$_3$-H$^+$]$^+$} 992.2017, found 992.2002.

3.6 Synthesis of enantiopure *Δ*-/*Λ*-MOC-16

(1) *Δ*-MOC-16. The enantiomer ***Δ*-3-PF$_6$** (10 mg, 0.008 mmol) and Pd$(BF_4)_2$ $(CH_3CN)_4$ (3 mg, 0.007 mmol) were dissolved in 1 mL of DMSO. The reaction mixture was kept at 80℃ for 3 h. 5 ml of ethyl acetate was added to the solution to form red precipitation immediately. After centrifugation, the solid was washed with 3 mL of ethyl acetate twice and dried under vacuum to give the desired product. Yield: 12 mg (90 %). ^{1}H NMR (400 MHz, DMSO-d_6 : D_2O = 1:5 v/v): δ 10.10 (s, 24H), 9.28 (s, 24H), 8.88 (d, J = 7.9 Hz, 24H), 8.79 (d, J = 7.6 Hz, 48H), 7.98 (d, J = 44.2 Hz, 48H), 7.84 (s, 24H), 7.53 (d, J = 30.3 Hz, 48H). ESI$^+$-MS: *m/z* calcd for $C_{432}H_{246}N_{120}Pd_6Ru_8$ { [(*Δ*-MOC-16)-18H$^+$]$^{+10}$ 856.4969, found 856.4943; *m/z* calcd for $C_{432}H_{247}N_{120}PF_6Pd_6Ru_8$ {[(*Δ*-MOC-16)-17H$^+$+PF$_6^-$]$^{+10}$ 871.0941, found 871.0949; *m/z* calcd for $C_{432}H_{245}N_{120}Pd_6Ru_8$ {[(*Δ*-MOC-16)-19H$^+$]$^{+9}$ 951.5513, found 951.5538; *m/z* calcd for $C_{432}H_{246}N_{120}PF_6Pd_6Ru_8$ {[(*Δ*-MOC-16)-18H$^+$+PF$_6^-$]$^{+9}$ 967.7704, found 967.7673. $[\alpha]^{30}$D = -266 °, c = 0.5, H_2O. The red octahedral single crystals suitable for X-ray diffraction were obtained by diffusion of isopropyl ether into the MeCN solution of 5 mg ***Δ*-MOC-16** and 2 mg ***S*-BINOL** for 2 weeks. Growth of ***Δ*-MOC-16** and ***R*-BINOL** co-crystals failed due to rapid precipitation upon diffusion of isopropyl ether.

(2) *Λ*-MOC-16. Compound ***Λ*-MOC-16** was prepared by the procedure detailed above for ***Δ*-MOC-16**. The enantiomer ***Λ*-3-PF$_6$** was used as starting material instead of compound ***Δ*-3-PF$_6$**. Yield: 12 mg (90 %). ^{1}H NMR (400 MHz, DMSO-d_6 : D_2O = 1:5 v/v): δ 10.13 (s, 24H), 9.29 (d, J = 5.2 Hz, 24H), 8.90 (d, J = 7.9 Hz, 24H), 8.80 (d, J = 7.8 Hz, 48H), 7.99 (d, J = 58.7 Hz, 48H), 7.85 (s, 24H), 7.62 – 7.45 (m, 48H). ESI$^+$-MS: *m/z* calcd for $C_{432}H_{245}N_{120}Pd_6Ru_8$ { [(*Λ*-MOC-16)-19H$^+$]$^{+9}$ 951.5513, found 951.5542; *m/z* calcd for $C_{432}H_{244}N_{120}Pd_6Ru_8$ {[(*Λ*-MOC-16)-20H$^+$]$^{+8}$ 1070.3693, found 1070.3677; *m/z* calcd for $C_{432}H_{243}N_{120}Pd_6Ru_8$ {[(*Λ*-MOC-16)-21H$^+$]$^{+7}$ 1223.1353, found 1223. 1334. $[\alpha]^{30}$D = 272 °, c = 0.5, H_2O. The red octahedral single crystals suitable for X-ray diffraction were obtained by diffusion of isopropyl ether into the MeCN solution of 5 mg ***Λ*-MOC-16** and 2 mg ***R*-BINOL** for 2 weeks. Growth of ***Λ*-MOC-16** and ***S*-BINOL** co-crystals failed due to rapid precipitation upon diffusion of isopropyl ether.

4. Chiral resolution of guest molecules by enantiopure *Δ*-/*Λ*-MOCs-16

4.1 Chiral resolution result for different guest molecules

(1) (±)-1,1'-Bi(2-naphthol) (BINOL)

As demonstrated in Supplementary Scheme 1, upon combination and vigorous stirring of an aqueous solution of ***Δ*- or *Λ*-MOC-16** (1 mM, 1 mL) and an ethereal layer of racemic BINOL (30 mg, 1 mL) at room temperature for 2 h, the bottom layer was taken out of the solution and extracted with $CHCl_3$ (3 × 4 mL). The extract was combined and the solvent was removed by rotary evaporator to afford white solid as resolved guest by chiral MOC host. The solid was redissolved with 0.5 mL isopropanol. The enantiomeric excess of BINOL was determined by HPLC (Chiralcel AD-H column, isopropanol/hexane = 15:85; flow rate 1.5 mL/min).

(2) (±)-3,3'-Dibromo-1,1'-bi-2-naphthol (3-Br-BINOL)

The resolution procedure was similar to the resolution of BINOL except for using 3-Br-BINOL as the starting materials. The enantiomeric excess of 3-Br-BINOL was determined by HPLC (Chiralcel AD-H column, isopropanol/hexane = 20:80; flow rate 1.0 mL/min).

(3) (±)-6,6'-Dibromo-1,1'-bi-2-naphthol (6-Br-BINOL)

The resolution procedure was similar to the resolution of BINOL except for using 6-Br-BINOL as the starting materials. The enantiomeric excess of 6-Br-BINOL was determined by HPLC (Chiralcel AD-H column, isopropanol/hexane = 15:85; flow rate 1.5 mL/min).

(4) (±)-1,1'-spirobiindane-7,7'-diol (Spirodiol)

The powder of Spirodiol (7.6 mg, 0.03 mmol) was suspended in the aqueous solution of ***Δ*- or *Λ*-MOC-16** (1 mM, 1 mL). The mixture was stirred at room temperature for 0.5 h. After centrifugation, the filtrate was collected and extracted with $CHCl_3$ (3 × 5 mL). The extract was combined and removed by rotary evaporator to afford white solid as resolved guest by chiral host. The solid was redissolved with 0.5 mL isopropanol. The enantiomeric excess of Spirodiol was determined by HPLC (Chiralcel AD-H colunm, isopropanol/hexane = 15:85; flow rate 1.0 ml/min).

(5) (±)-2-(6-Methoxynaphthalen-2-yl)propanoic acid (Naproxen)

Upon combination and vigorous stirring of an aqueous solution of ***Δ*- or *Λ*-MOC-16** (1 mM, 1 mL) and an ethereal layer of racemic Naproxen (15 mM, 2 mL) at room temperature for 0.5 h,

the bottom layer was taken out of the solution and extracted with $CHCl_3$ (3 × 4 mL). The extract was combined and the solvent was removed by rotary evaporator to afford white solid as resolved guest by chiral host. The solid was redissolved with 0.4 mL isopropanol. The enantiomeric excess of Naproxen was determined by HPLC (Chiralcel AS-H column, isopropanol/hexane/TFA = 8:92:0.1; flow rate 1.0 mL/min).

(6) (±)-1-(1-Naphthyl)ethanol

The powder of 1-(1-Naphthyl)ethanol (5.2 mg, 0.03 mmol) was suspended in the aqueous solution of ***Δ*- or *Λ*-MOC-16** (1 mM, 1 mL). The mixture was stirred at room temperature for 0.5 h. After centrifugation, the filtrate was collected and extracted with $CHCl_3$ (3 × 5 mL). The extract was combined and removed by rotary evaporator to afford white solid as resolved guest by chiral host. The solid was redissolved with 0.5 mL isopropanol. The enantiomeric excess of 1-(1-Naphthyl)ethanol was determined by HPLC (Chiralcel OD-H column, isopropanol/hexane=10:90; flow rate 1.0 mL/min).

(7) (±)-2-Hydroxy-2-phenylacetophenone (Benzoin)

The powder of Benzoin (6.4 mg, 0.03 mmol) was suspended in the aqueous solution of ***Δ*-** or ***Λ*-MOC-16** (1 mM, 1 mL). The mixture was stirred at room temperature for 0.5 h. After centrifugation, the filtrate was collected and extracted with $CHCl_3$ (3 × 5 mL). The extract was combined and removed by rotary evaporator to afford white solid as resolved guest by chiral host. The solid was redissolved with 0.5 mL isopropanol. The enantiomeric excess of Benzoin was determined by HPLC (Chiralcel OD-H column, isopropanol/hexane = 10:90; flow rate 1.5 mL/min).
